# Heart rate variability measures indicating sex differences in autonomic regulation during anxiety-like behavior in rats

**DOI:** 10.3389/fpsyt.2023.1244389

**Published:** 2023-10-31

**Authors:** Raizel M. Frasier, Thatiane De Oliveira Sergio, Phillip A. Starski, Angela J. Grippo, F. Woodward Hopf

**Affiliations:** ^1^Department of Psychiatry, Indiana University School of Medicine, Indianapolis, IN, United States,; ^2^Medical Scientist Training Program, Indiana University School of Medicine, Indianapolis, IN, United States; ^3^Department of Psychology, Northern Illinois University, DeKalb, IL, United States; ^4^Stark Neurosciences Research Institute, Indiana University, Indianapolis, IN, United States

**Keywords:** autonomics, heart rate variability, anxiety, sex differences, sympathetic, parasympathetic

## Abstract

**Introduction:**

Mental health conditions remain a substantial and costly challenge to society, especially in women since they have nearly twice the prevalence of anxiety disorders. However, critical mechanisms underlying sex differences remain incompletely understood. Measures of cardiac function, including heart rate (HR) and HR variability (HRV), reflect balance between sympathetic (SNS) and parasympathetic (PNS) systems and are potential biomarkers for pathological states.

**Methods:**

To better understand sex differences in anxiety-related autonomic mechanisms, we examined HR/HRV telemetry in food-restricted adult rats during novelty suppression of feeding (NSF), with conflict between food under bright light in the arena center. To assess HRV, we calculated the SDNN (reflective of both SNS and PNS contribution) and rMSSD (reflective of PNS contribution) and compared these metrics to behaviors within the anxiety task.

**Results:**

Females had greater HR and lower SNS indicators at baseline, as in humans. Further, females (but not males) with higher basal HR carried this state into NSF, delaying first approach to center. In contrast, males with lower SNS measures approached and spent more time in the brightly-lit center. Further, females with lower SNS indicators consumed significantly more food. In males, a high-SNS subpopulation consumed no food. Among consumers, males with greater SNS ate more food.

**Discussion:**

Together, these are congruent with human findings suggesting women engage PNS more, and men SNS more. Our previous behavior-only work also observed female differences from males during initial movement and food intake. Thus, high basal SNS in females reduced behavior early in NSF, while subsequent reduced SNS allowed greater food intake. In males, lower SNS increased engagement with arena center, but greater SNS predicted higher consumption. Our findings show novel and likely clinically relevant sex differences in HRV-behavior relationships.

## Introduction

Mental health conditions remain a substantial challenge to society, where anxiety and mood conditions alone extract approximately 700 billion US dollars per year globally (1). Additionally, epidemiological data reveal that women have nearly twice the prevalence of anxiety disorders compared to men (2–6). Given that sex differences exist, it is of considerable importance to investigate the possible biological underpinnings that influence such differences, which would then facilitate the development of sex-specific and individualized treatment options. Currently, critical mechanisms that underly observed sex differences in anxiety-related conditions remain incompletely understood. Furthermore, in the study of humans, it is often challenging to truly disambiguate biological versus socio-cultural contributions to psychopathologies, including anxiety disorders (2, 5, 6). Thus, utilizing rodent models of anxiety-like behavior is of immense value, since such models allow for the control necessary to reveal mechanistic insights into observed sex differences.

Investigations of sex differences in anxiety-like responding in rodent models have been performed by many groups (3, 4, 6–9) including our lab (8). Such studies have shown that females typically locomote more (10, 11) and have differential responding patterns to stressors compared to males, where females will display “darting” or avoidance behavior, whereas males will display more freezing behavior (3, 4, 6, 7). To complicate matters, nearly all anxiety-like behavior paradigms for rodents were initially developed and validated using only males (3, 4, 6, 7). However, by assessing multiple measures within a given anxiety-like paradigm, one can potentially gain insights into behavioral sex differences, as our group previously showed using the Novelty Suppression of Feeding (NSF) task (detailed further below) (8).

**Figure 1:**
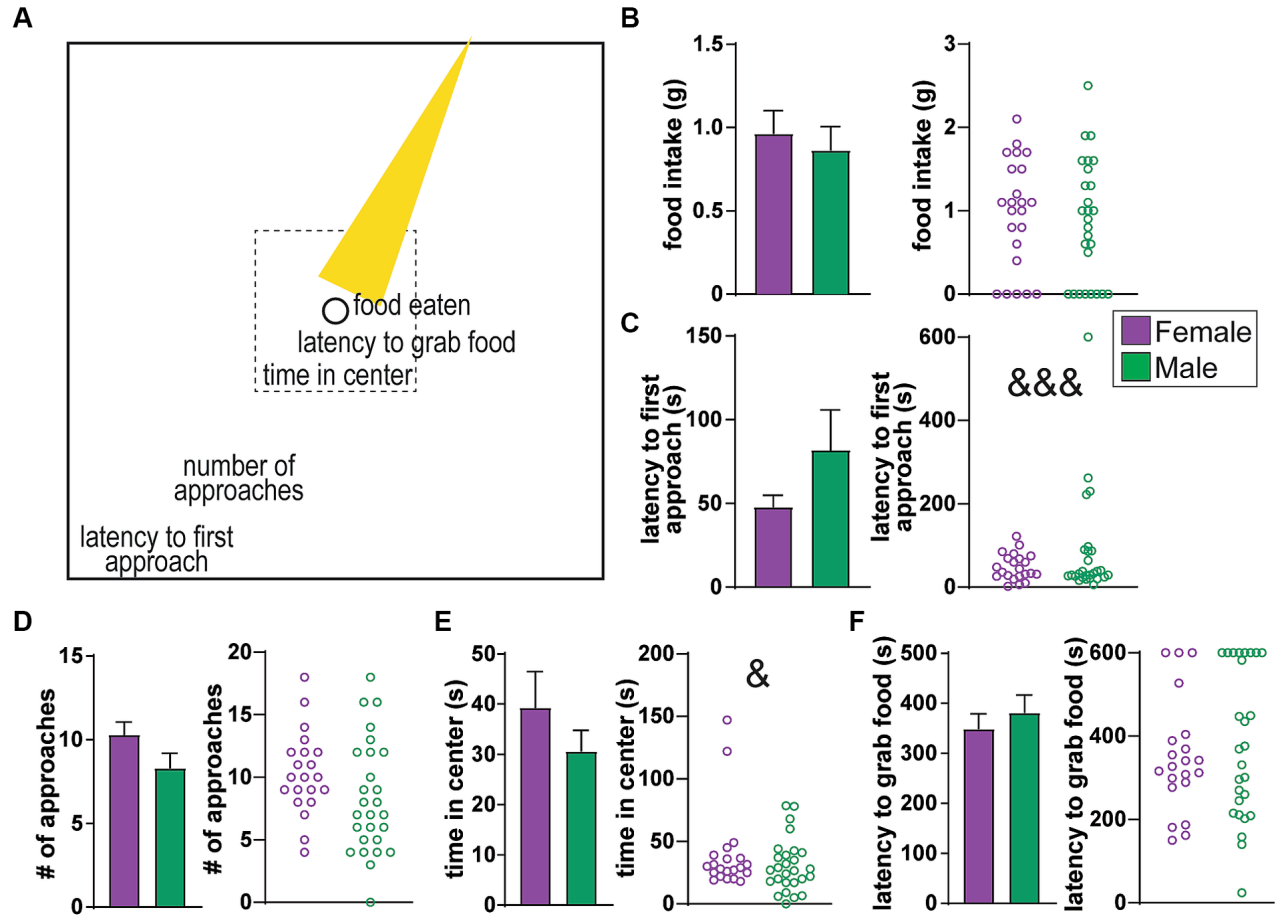
No sex differences in behavioral measures of the Novelty Suppression of Feeding (NSF) task. **(A)** Cartoon of the NSF arena, where a food-restricted rat has access to food in the center of the arena (circle) which is brightly lit (yellow cone). The task consists of a series of progressive measures before food consumption, including latency to first approach, the number of approaches, time in center (tolerating the light), and latency to grab the food. **(B)** Food intake. **(C)** Latency to first approach the center. **(D)** Number of approaches. **(E)** Time in center. **(F)** Latency to grab food. No average differences, although variability of latency to approach was greater in females (&&& *p <* 0.001), and variability of time in center was greater in males (& *p <* 0.05).

The NSF task consists of restricting the rat’s food intake for 3 days prior to placement in a novel chamber which has food in the center under a bright light. The NSF task leverages the conflict between the hungry rat’s drive to feed and its innate aversion to brightly lit and open spaces. One benefit of the NSF task is that there are many progressive behavioral measures prior to food consumption, such as (A) the latency to first approach the brightly lit center, (B) the number of approaches to the center, (C) the time spent in the center, (D) the latency to grab the food, and, finally, (E) the amount of food consumed during the task (illustrated in Figure 1A). Our group recently demonstrated that females displayed greater anxiety-like behavior related to food acquisition in NSF (greater latency to grab food and less food consumed) compared to males, using a smaller NSF arena (8) than utilized here. We posited that these results suggest female anxiety-like behavior is increased in relation to specific, life-relevant situations, such as obtaining food when hungry in the presence of aversions.

To better understand the mechanistic basis of any sex differences in anxiety-like behaviors, our lab sought to elucidate potential physiologic underpinnings by using cardiovascular telemetry to determine autonomic state. Recently, there has been increased excitement in the use of cardiac measures, including heart rate (HR) and HR variability (HRV), as intriguing candidates for biomarkers of and/or contributors to pathological states, especially in the realm of mental health conditions (12–14). HR and HRV are modulated by a dynamic balance between the two opposing divisions of the autonomic nervous system: the sympathetic (SNS, “fight or flight”) and the parasympathetic (PNS, “rest and digest”) (13–16). HR, the number of beats per minute, is straightforward to both measure and interpret. In contrast, there are multiple methods used to calculate HRV, which elucidate different aspects of autonomic influences on the heart.

Much prior work has been performed to assess and validate how particular HRV measures reflect different aspects of autonomic state, especially the relative influence of SNS and PNS. Drugs that target specific aspects of cardiac physiology have been especially valuable.

For example, beta-adrenergic receptors are known to mediate SNS influences on the heart, and thus one can use the effects of beta-adrenergic receptor blockers (such as propranolol) on HRV to gain insight into the relative influence of SNS on a given HR/HRV measure. In parallel, muscarinic acetylcholine receptors are known to contribute to PNS impacts, and by using a selective blocker (such as atropine), one can better isolate PNS influences on heart measures. Indeed, such studies have been done in humans (17, 18), dogs (19, 20), and rat (16, 21, 22) to demonstrate the validity of HRV indicators to discern autonomic tone.

**Figure 2:**
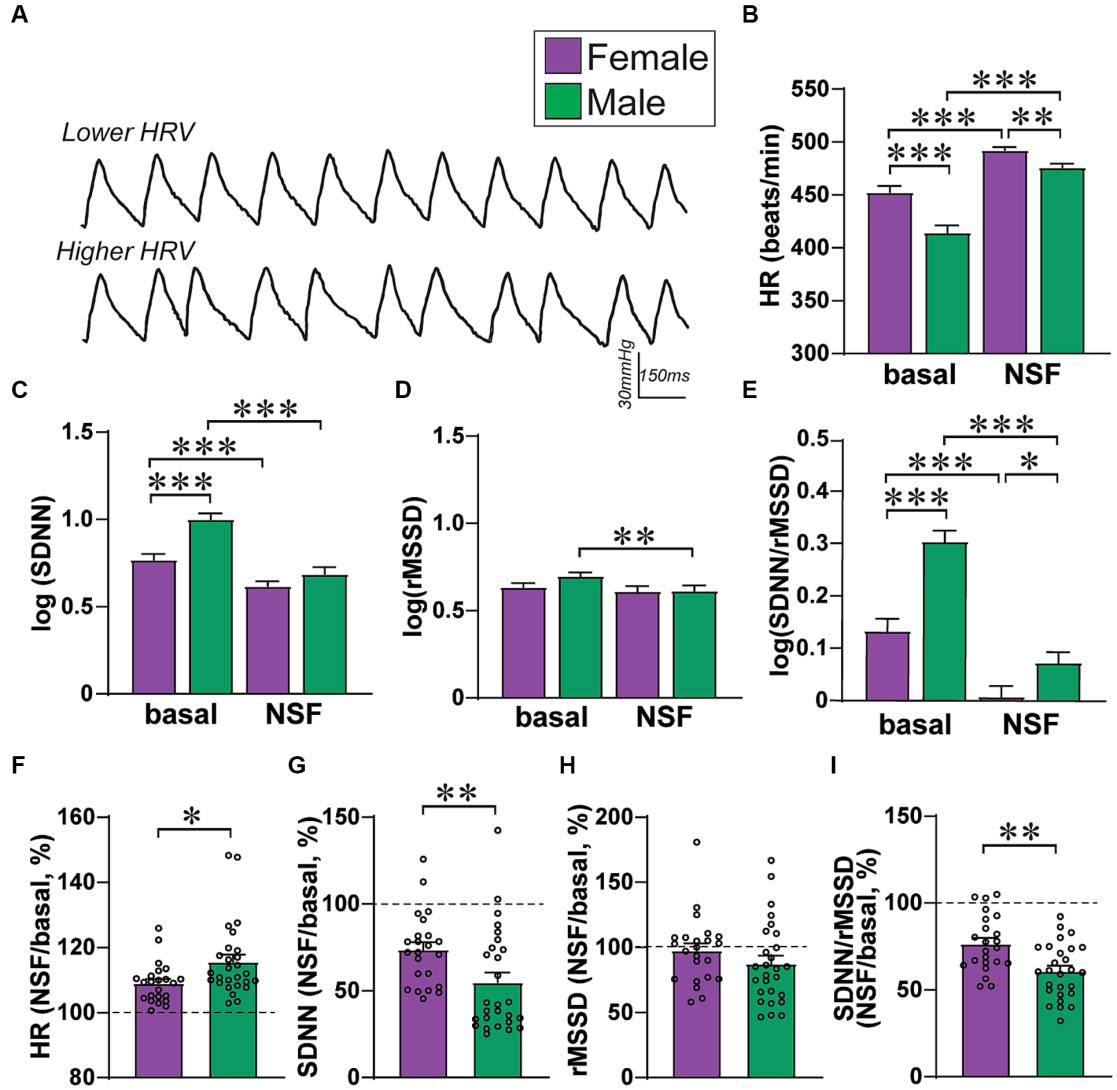
Female–male differences in basal HR/HRV measures. **(A)** Examples of HR traces with lower HRV (top) and higher HRV (bottom, exaggerated cartoon version). **(B–E)** Relative to males, females had **(B)** greater HR, **(C)** lower SDNN, **(D)** no rMSSD differences, and **(E)** lower SDNN/rMSSD ratio. To perform two-way ANOVA, data were log-normalized for SDNN, rMSSD, and SDNN/rMSSD. **(F–I)** Males show larger baseline-to-NSF changes in several HRV measures, when calculating percent change in a given measure within-rat. *,**,*** *p <* 0.05, *p <* 0.01, *p <* 0.001.

**Figure 3:**
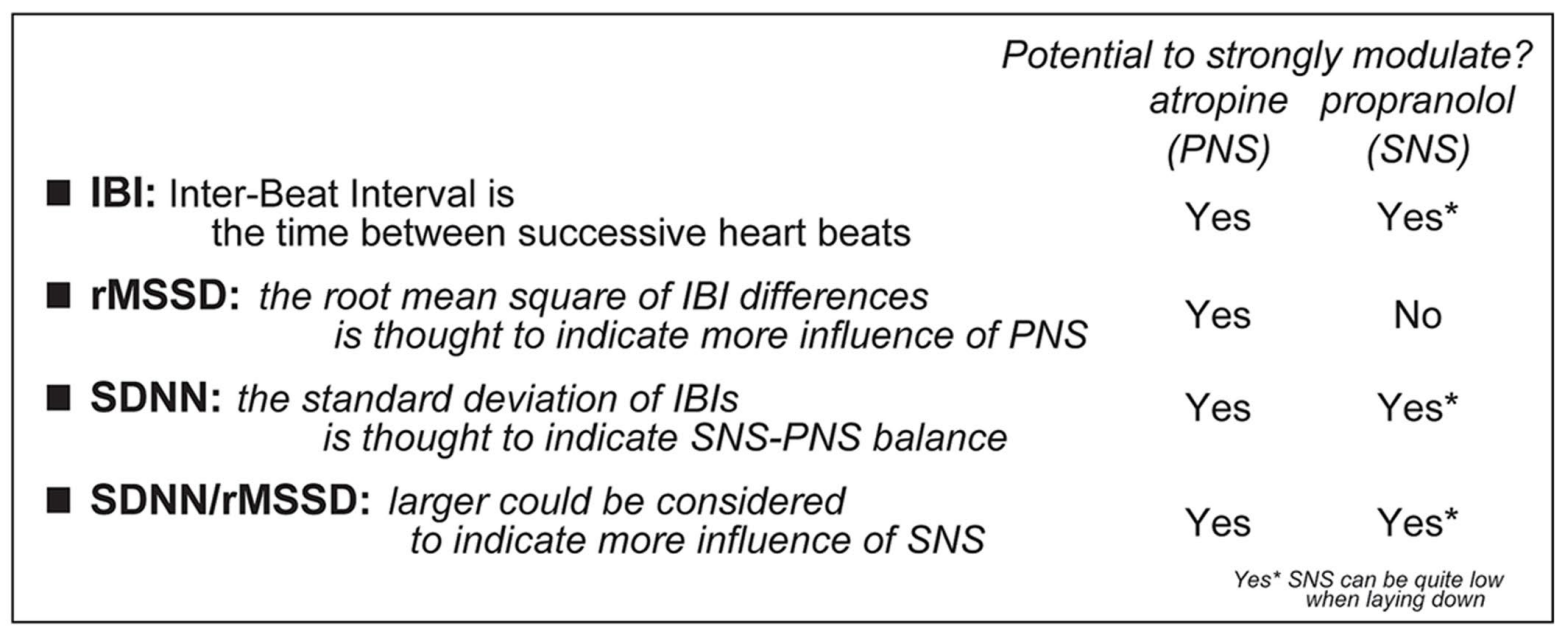
Description of how to calculate different HRV measures, including how they could relate to PNS and/or SNS. Specifically, some pharmacological agents are considered to impact the SNS, through the beta-adrenergic receptors (which are blocked by propranolol), or the PNS, through the muscarinic cholinergic receptors (which are blocked by atropine) [this interpretation does require caution since laying down is related to low SNS (17, 18, 23, 24)].

Here, we focus on two widely-used measurements of HRV, where we assess patterns of heart rate variability across a series of inter-beat intervals, the amount of time between successive heartbeats (these are called “time domain” measures”). Examples of HR traces are shown in Figures 2A, 3 summarizes the HRV measures used here and how they are impacted by the drugs described above. First, we examined the rMSSD, the root Mean Squared of the Successive Differences in inter-beat intervals (IBIs), is altered by atropine but not propranolol, and thus is considered to primarily reflect PNS influence rather than SNS. SDNN, which is the standard deviation of a series of inter-beat intervals (“NN” is the time between the peaks of two heartbeats), is altered by either propranolol or atropine. Thus, SDNN is considered to indicate a balance between SNS and PNS influences on the heart. Finally, by taking the ratio of SDNN/rMSSD, we can discern the relative impact of the SNS, since this ratio is effectively [SNS influence + PNS influence]/[PNS influence]. Thus, greater ratio is taken to indicate greater SNS influence (13–16, 25, 26).

A series of related measures examine HRV in the frequency domain, with the high-frequency component (HFHRV) related to PNS (similar to rMSSD), and the low-frequency (LFHRV) related to PNS-SNS balance (similar to SDNN) (27–30). Recent work has considered rMSSD and SDNN of high utility, in part due to analytic differences across studies of frequency-related indicators (18, 26, 31). For example, one study (32) found reduced rMSSD but not HFHRV in alcohol drinkers, which they suggest might reflect analytic differences from other studies finding altered HFHRV. Another recent study chose rMSSD for a large meta-analysis of sex differences in HRV (26). Nonetheless, rMSSD often correlates well with HFHRV, and SDNN with LFHRV (25, 26). Thus, we focus here on SDNN, rMSSD, and SDNN/rMSSD. By assessing across HRV measures, one can gain insight into underlying autonomic changes as a function of sex and behavioral condition.

Since HRV measures can reliably assess several aspects of autonomic state, it is thus a useful tool for the study of sex differences in autonomic regulation, especially in mental health conditions. Indeed, various studies have found sex differences in autonomic regulation, where women have higher HR than men under various conditions, including at baseline (26, 31, 33). However, while one might think that a higher HR would automatically result in lower HRV (since less time between heartbeats leaves less opportunity for variability, mathematically), paradoxically, women tend to display a higher baseline HRV than men even though they have greater HRs (26, 31, 33, 34). For example, in a large recent study (31), women had greater HRV than men for a given HR level, while on average women had lower SDNN but no differences in rMSSD (31, 34). Furthermore, in addition to these sex differences in basal HR and HRV, several studies have demonstrated sex differences in autonomic tone during challenge, where women tend to recruit PNS more, and, in contrast, men tend to recruit SNS more (15, 26, 34–36). Other studies also suggest sex differences in autonomic regulation at the level of different brain circuits (34, 37–40). However, the functional implications of such sex differences on behavior are understudied, and much remains unknown about how autonomic measures relate to specific aspects of anxiety-like behavior, especially potential sex differences in HR/HRV measures during expression of anxiety-like behavior.

We note that the present studies include data collected from both naïve and alcohol-drinking rats. In humans, alcohol drinkers can often show autonomic differences relative to controls (13, 41). Thus, initially we had hypothesized there would be differences in autonomic tone not only by sex, but also by rat drinking history (e.g., “alcohol-naïve” versus near daily “alcohol-drinking” rats). However, we found no such differences in NSF behavior or their autonomic correlates in naives vs. drinkers (Supplementary Figures 1, 2); we further explore the potential reasons for these findings in the Discussion. Thus, for the current study, we collapsed data by drinking history and instead presented the data by sex, to increase the sample size and maximize the ability to observe HR/HRV differences across sex and different anxiety-like behavior measures.

As such, the present study is the first, to our knowledge, to have used cardiovascular telemetry to assess HR and HRV indicators in adult female and male Wistar rats in relation to different behavioral measures within the NSF anxiety-like behavior task. We also compared NSF behavioral measures to HR/HRV at baseline, defined as the period of time immediately preceding the NSF task. Overall, we found significant sex differences in cardiac autonomic regulation that related to different NSF behaviors. In females, HRV measures predicted food intake and the first approach in the task, and our previous studies (8) suggest that food consumption and the first movement in a novel context are of particular relevance to females. In males, HRV patterns predicted different NSF behaviors than in females, for example, where greater SNS indicators correlated with greater male intake in males who consumed food, the opposite of females. Finally, females had baseline autonomic differences that persisted into the anxiety testing period and impacted behavior, while male NSF behavior related to HRV measures within the task but not at baseline. Together, our results uncovered several important and novel sex differences in autonomic influences over anxiety-like responding, which may provide both biomarkers for and mechanistic insights into sex differences in anxiety mechanisms.

## Materials and methods

### Animals

All experiments were performed in accordance with NIH Guidelines and approved by the Institutional Animal Care and Use Committee (IACUC) of Indiana University. Post-natal day ~50–55 male and female Wistar rats (Envigo) were singly housed in clear plastic homecages with *ad libitum* food and water in a 12 h reverse light cycle (lights off 8 AM), and with both sexes in the same housing rooms. At the time of anxiety testing, rats were 6–7 month old alcohol-naïve or alcohol drinkers (see Supplementary Methods). However, we found no differences in NSF behavior or in nearly all HRV measures between drinkers and alcohol naïve rats (Supplementary Figures 1, 2). On the other hand, we did observe greater HR and lower SDNN in females versus males (in both drinkers and naives), similar to what is seen for female and male humans (31) and other rat strains (42), making it likely that our telemetry is robustly assessing autonomic function. Thus, we collapsed results from drinkers and naives for all other analyses. A further explanation on plausible reasons for the lack of influence of drinking history on NSF behavior and HR/HRV measures is detailed in the Discussion.

In addition, we note that the intermittent access alcohol model we use exhibits features related to human drinking, including escalating intake (43, 44), sensitivity to compounds that reduce human drinking (44–48), withdrawal symptoms (although moderate) (49, 50), and front loading, where strong initial drinking suggests high motivation for alcohol (51–53). Importantly, long-drinking Wistars also show compulsion-like intake, where drinking persists despite negative consequence (45, 46, 48) that is, at least in part, mediated through an insula circuit (51, 54) that somewhat similar to that observed during problem drinking in humans (55, 56). Thus, while alcohol drinking did not produce human-like changes in HRV (which we consider further in Supplementary Figure 2 legend), the drinking model we use likely captures several aspects of human addiction.

### Anxiety-like behavior

Studies occurred in a self-standing custom-built soundproof chamber (6 ft. by 8 ft. base, 7 ft. high) with ventilation fans to minimize disturbance during testing. The behavioral arena was cleaned between rats with 0.025% bleach, and 15-20 min air dry. Rats underwent Novelty Suppression of Feeding (NSF), with behavior videotaped for later scoring by an observer blind to the experimental condition. For NSF, rats were food restricted for 3 days. Intake was determined for ~4 days before food restriction, to determine average intake for each rat. Rats then received 80% of their normal food intake for two days, and 20% of normal intake for a third day. NSF was tested the following morning in a single 10-min session. A 10-min baseline session was recorded in the home cage immediately preceding NSF testing. The NSF arena was square (100cm × 100cm) with 40 cm high walls, custom made from black acrylic plastic. During the NSF test, a bright light shines at the center, targeting ~3 g food on a 3″ circular white paper (160lux at center, <60lux along edges). After all rats were run in NSF, they were given a minimum 5 min in their home cage before being tested in the Light–Dark Box. Here, we present only findings from NSF. Light–Dark box analyses are ongoing, and we have considered NSF as our main task of interest, with conflict between bright light and food, while light–dark box only involves aversion to bright light (a control). NSF measures were the same as we previously assessed (8) (Figure 1A), and included latency to first approach the center, number of approaches to center, time in center, latency to first grab food, and food eaten. Latency to eat is a widely used measure for NSF tasks, without sex differences, even with some differences in NSF methods (57–60). The complexity of the stressor is an advantage since it is likely to reliably and fully engage the integrated systems of interest here.

### Telemetry, HR, and HRV analyses

Surgery is in Supplementary Methods, implanting a Stellar telemetry device (type PTA-M-C, part# E-430001-IMP-130) from TSE Systems Inc. (Chesterfield, MO). After 5–7 days of recovery from surgery, rats began food restriction. Real-time blood pressure traces were recorded using NOTOCORD-hem software (Instem, Staffordshire, United Kingdom), with all baseline data recorded in the home cage before NSF. The NOTOCORD-hem software recorded blood pressure changes at 250 Hz, with pressure fluctuations related to each heartbeat. Here, we focus on HR and HRV, as determining the actual absolute blood pressure values might be more challenging relative to assessing HR.

Occasionally there were artifacts and missed heartbeats. Thus, each raw 10-min recording was visually inspected to confirm each heartbeat was correctly detected, and to remove such data to ensure accurate reporting of cardiovascular measures. This is similar to what is typically performed in many other studies (42, 61–63), and was typically less than 2% of a session. The NOTOCORD-hem software provided the timing of each heartbeat peak. We used this time series to calculate overall HR (beats/min) and to determine inter-beat intervals (IBI, in msec) across the session. Using IBIs, the following time-domain HRV values were determined: (1) SDNN, the standard deviation of the IBIs for a given period of time, reflecting both SNS and PNS influences, and (2) rMSSD, the root mean squared of the successive differences in IBI, which reflects PNS influence (Figure 3). rMSSD was determined by taking the square of difference between successive IBI values, averaging the squares of these differences, then taking the square root of this average. We also examined the SDNN/rMSSD ratio, which provides insight into the relative SNS contribution. Importantly, recent work in humans (31) suggests the importance of not adjusting HRV measures for HR, which results in missing important sex differences.

**Figure 4:**
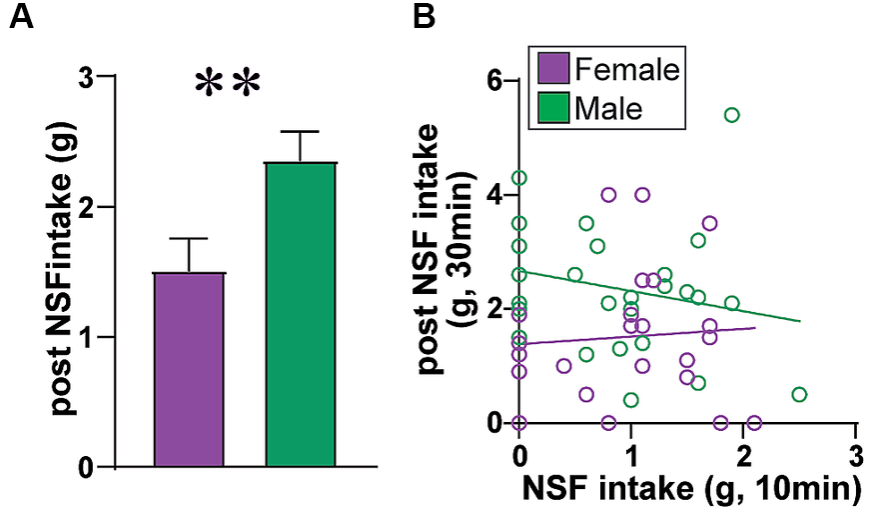
Males eat more food after NSF. Because the NSF is a brief test (10 min), amount of food consumed is typically not corrected for body weight. In support of this, rats had 30 min to eat food in the home cage after the NSF and LDB testing, and **(A)** male rats ate significantly more food in the post-NSF intake period than females (MW = 172.5, *p* = 0.0065), as seen previously (5). **(B)** Also, if rat intake in the 10 min NSF task was titrated by body weight and nutritional need, one might predict that rats that ate more during NSF would eat less in the later home-cage intake period. However, NSF intake and later home cage intake were not correlated in females (*F*_(1, 21)_=0.118, *R*^2^ = 0.006, *p =* 0.7348) or males (*F*_(1, 25)_=1.328, *R*^2^ = 0.050, *p =* 0.2601). Also, females had a trend for greater intake post vs. during NSF (Wilcoxon matched-pairs signed-rank test, diff in medians = 0.600, *p =* 0.0633), while males had significantly more intake post vs. during NSF (Wilcoxon, diff in medians = 1.300, *p <* 0.0001). Thus, it is reasonable to conclude that food intake during NSF was small enough that it does not need to be corrected to body weight, supporting the interpretation that there was no sex difference in intake level during the NSF task.

**Figure 5:**
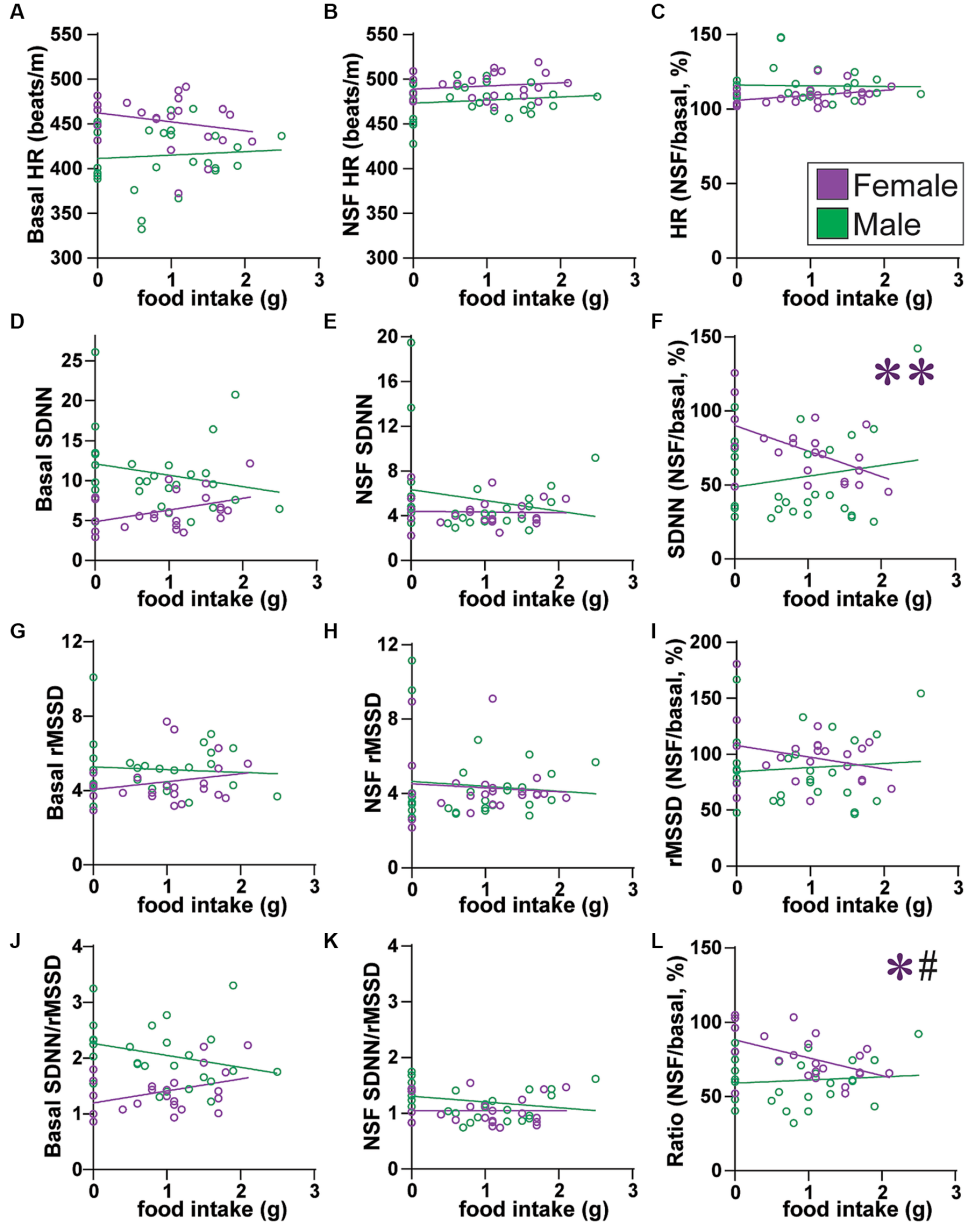
Across all rats, females with lower SNS indicators ate more food. **(A–C)** Food intake did not correlate with HR **(A)** at baseline (females: *F*_(1, 21)_ = 1.180, *R*^2^ = 0.053, *p =* 0.2897; males: *F*_(1, 25)_ = 0.158, *R*^2^ = 0.006, *p =* 0.6946), **(B)** during NSF (females: *F*_(1, 21)_ = 0.554, *R*^2^ = 0.026, *p =* 0.4651; males: *F*_(1, 25)_ = 0.472, *R*^2^ = 0.019, *p =* 0.3984), or **(C)** change from baseline to NSF period (females: *F*_(1, 21)_ = 2.944, *R*^2^ = 0.123, *p =* 0.1009; males: *F*_(1, 25)_ = 0.021, *R*^2^ = 0.001, *p =* 0.8851). **(D–F)** Food intake did not correlate with SDNN **(D)** at baseline (males: *F*_(1, 25)_ = 1.225, *R*^2^ = 0.047, *p =* 0.2789) or **(E)** during NSF (males: *F*_(1, 25)_ = 0.975, *R*^2^ = 0.038, *p =* 0.3330), but **(F)** females with larger drop in SDNN from baseline to NSF ate more food (males: *F*_(1, 25)_ = 0.824, *R*^2^ = 0.032, *p =* 0.3728). **(G–I)** Food intake did not correlate with rMSSD **(G)** at baseline (females, *F*_(1, 21)_ = 1.160, *R*^2^ = 0.052, *p =* 0.2938; males, *F*_(1, 25)_ = 0.146, *R*^2^ = 0.006, *p =* 0.7057), **(H)** during NSF (females, *F*_(1, 21)_ = 0.153, *R*^2^ = 0.007, *p =* 0.6992; males, *F*_(1, 25)_ = 0.228, *R*^2^ = 0.009, *p =* 0.6373), or **(I)** change from baseline to NSF period (females: *F*_(1, 21)_ = 1.547, *R*^2^ = 0.069, *p =* 0.2273; males: *F*_(1, 25)_ = 0.179, *R*^2^ = 0.007, *p =* 0.6759). **(J–L)** Food intake did not correlate with SDNN/rMSSD **(J)** at baseline (females: *F*_(1, 21)_ = 3.404, *R*^2^ = 0.140, *p =* 0.0792; males: *F*_(1, 25)_ = 2.197, *R*^2^ = 0.081, *p =* 0.1508) or **(K)** during NSF (females: *F*_(1, 21)_ = 0.000, *R*^2^ = 0.000, *p =* 0.9951; males: *F*_(1, 25)_ = 1.718, *R*^2^ = 0.064, *p =* 0.2019), but **(L)** females with larger drop in SDNN/rMSSD from baseline to NSF ate more food. Related correlations of log-transformed HR/HRV measures are shown in Supplementary Figure 10. *,** *p <* 0.05, *p <* 0.01. # <0.05 female–male difference in slopes.

**Figure 6:**
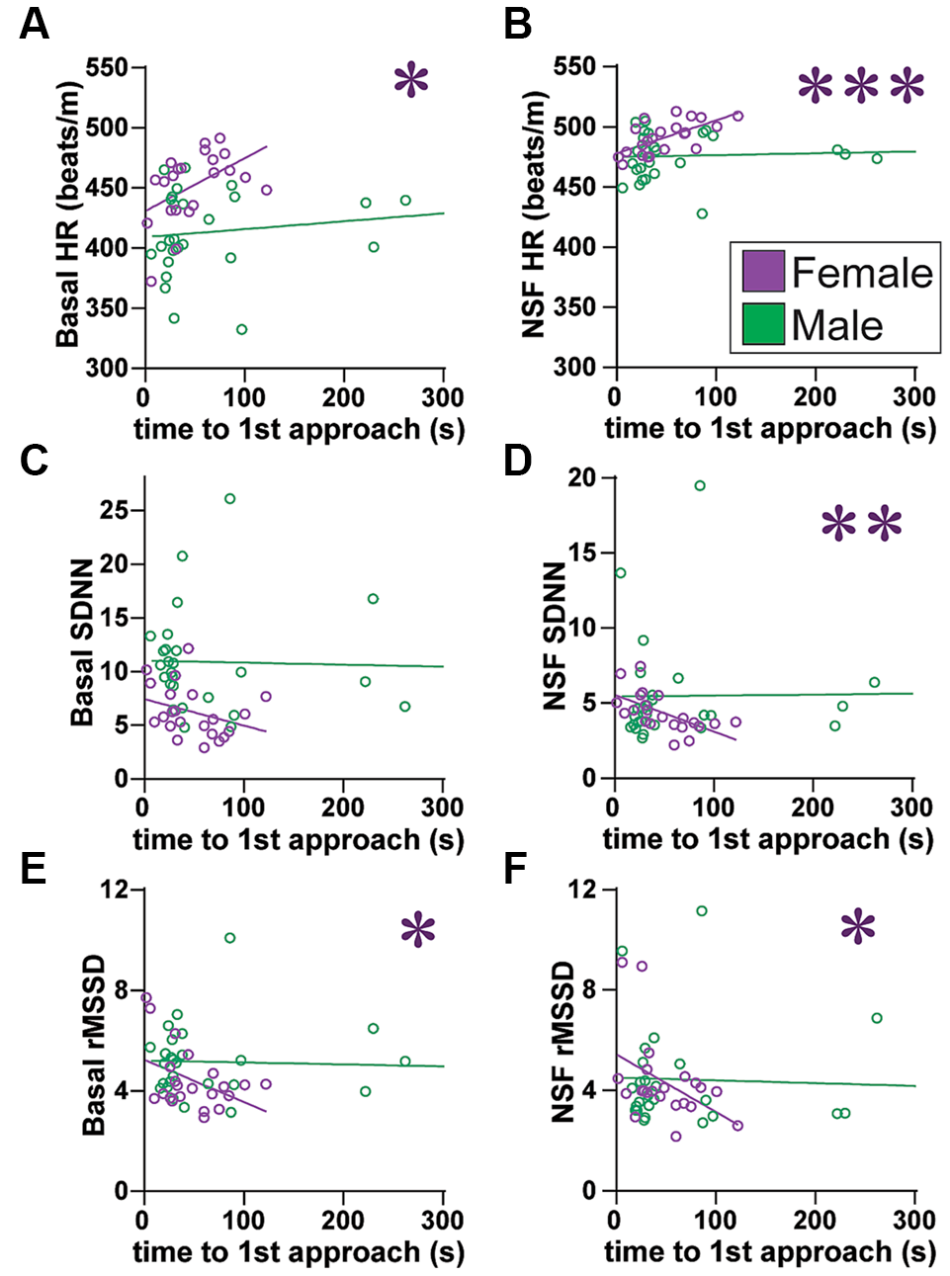
Females (but not males) with higher SNS indicators, including greater HR at baseline, had slower latency to first approach. **(A,B)** Females with higher HR **(A)** at baseline or **(B)** during NSF had significantly slower initial latency to approach the center (males: basal: *F*_(1, 25)_ = 1.413, *R*^2^ = 0.053, *p =* 0.2458; NSF: *F*_(1, 25)_ = 0.264, *R*^2^ = 0.010, *p =* 0.6118). **(C)** While basal SDNN did not correlate with slower latency (males: *F*_(1, 25)_ = 0.059, *R*^2^ = 0.002, *p =* 0.8099), **(D)** females with lower SDNN during NSF had slower initial approach (males: *F*_(1, 25)_ = 0.011, *R*^2^ = 0.001, *p =* 0.9175). **(E,F)** Females with lower rMSSD **(E)** at baseline or **(F)** during NSF had significantly slower initial latency to approach center [males: **(E)**, *F*_(1, 25)_ = 0.114, *R*^2^ = 0.114, *p =* 0.7386; **(F)**, *F*_(1, 25)_ = 0.115, *R*^2^ = 0.005, *p =* 0.7373]. No HRV measures related to male latency. Related correlations of log-transformed HR/HRV measures are shown in Supplementary Figure 11. *,**,*** *p <* 0.05, *p <* 0.01, *p <* 0.001.

**Figure 7:**
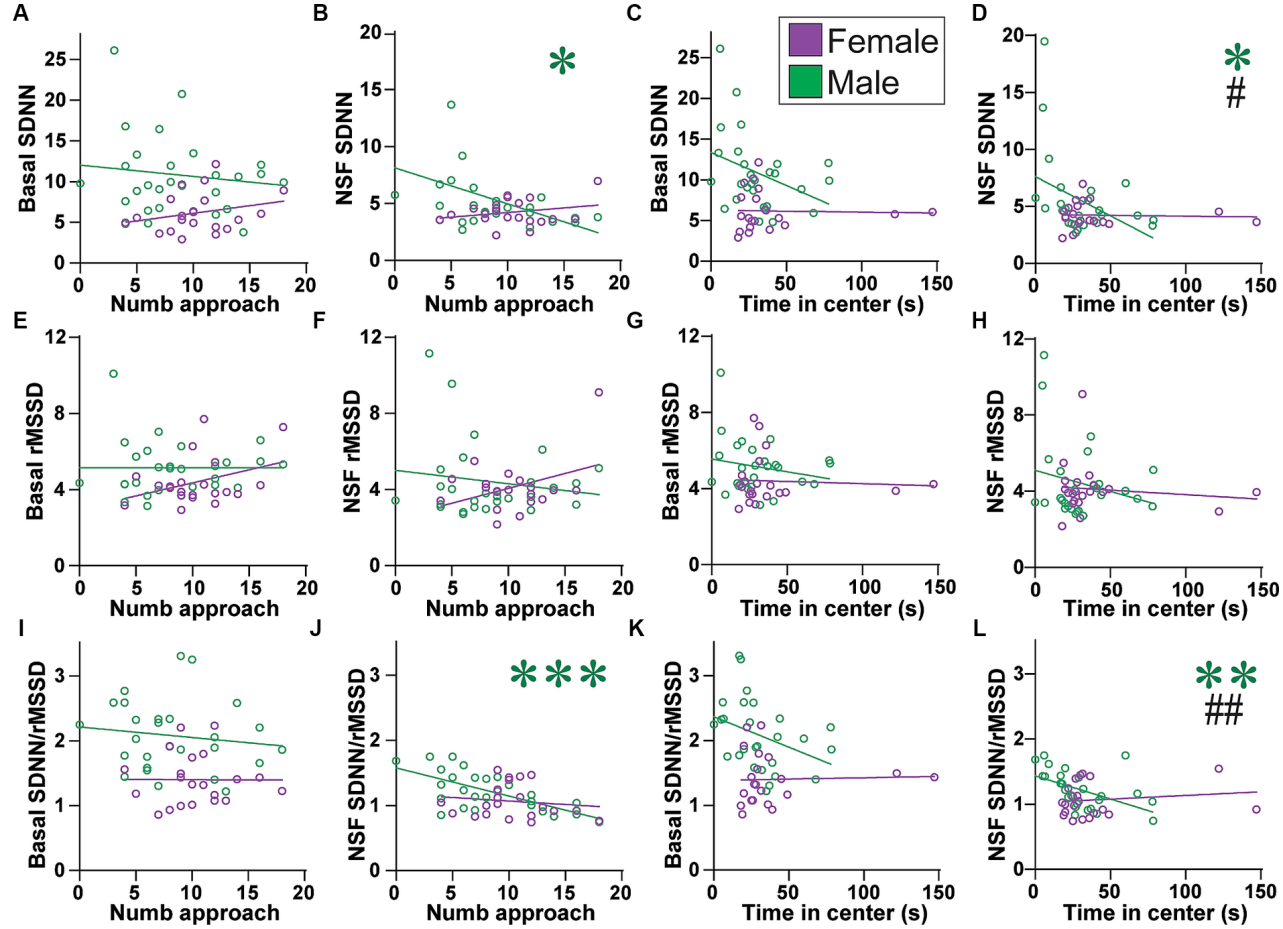
Males (but not females) with lower SNS indicators had more approaches to and time in center. **(A–D)** While basal SDNN did not correlate with **(A)** number of approaches (males: *F*_(1, 25)_ = 0.433, *R*^2^ = 0.017, *p =* 0.5167; females: *F*_(1,19)_ = 1.319, *R*^2^ = 0.065, *p =* 0.2650) or **(C)** time in center (females: *F*_(1, 19)_ = 0.017, *R*^2^ = 0.000, *p =* 0.8986), males with lower SDNN during NSF had **(B)** more approaches and **(D)** more time in center. **(E–H)** rMSSD did not correlate any measure, at baseline **(E,G)** or during NSF **(F,H)**, or for **(E,F)** number of approaches or **(G,H)** time in center. **(E)**, males: *F*_(1, 25)_ = 0.000, *R*^2^ = 0.000, *p =* 0.9964; females: *F*_(1, 19)_ = 2.800, *R*^2^ = 0.128, *p =* 0.1107; **(F)**, males: *F*_(1, 25)_ = 0.641, *R*^2^ = 0.025, *p =* 0.4309; females: *F*_(1, 19)_ = 3.212, *R*^2^ = 0.145, *p =* 0.0891; **(G)**, males: *F*_(1, 25)_ = 1.031, *R*^2^ = 0.040, *p =* 0.3196; females: *F*_(1, 19)_ = 0.071, *R*^2^ = 0.004, *p =* 0.7925; **(H)**, NSF: males: *F*_(1, 25)_ = 1.421, *R*^2^ = 0.054, *p =* 0.2444; females: *F*_(1, 19)_ = 0.258, *R*^2^ = 0.013, *p =* 0.6171). **(I–L)** While basal SDNN/rMSSD did not correlate with **(I)** number of approaches (males: *F*_(1, 25)_ = 0.454, *R*^2^ = 0.018, *p =* 0.5065; females: *F*_(1, 19)_ = 0.001, *R*^2^ = 0.000, *p =* 0.9784) or **(K)** time in center (females: *F*_(1, 19)_ = 0.022, *R*^2^ = 0.001, *p =* 0.8824), males with lower SDNN/rMSSD during NSF had **(J)** more approaches and **(L)** more time in center. Related correlations of log-transformed HR/HRV measures are shown in Supplementary Figures 12, 13. *,**,*** *p <* 0.05, *p <* 0.01, *p <* 0.001. #,## *p <* 0.05, *p <* 0.01 female–male difference in slopes.

**Figure 8:**
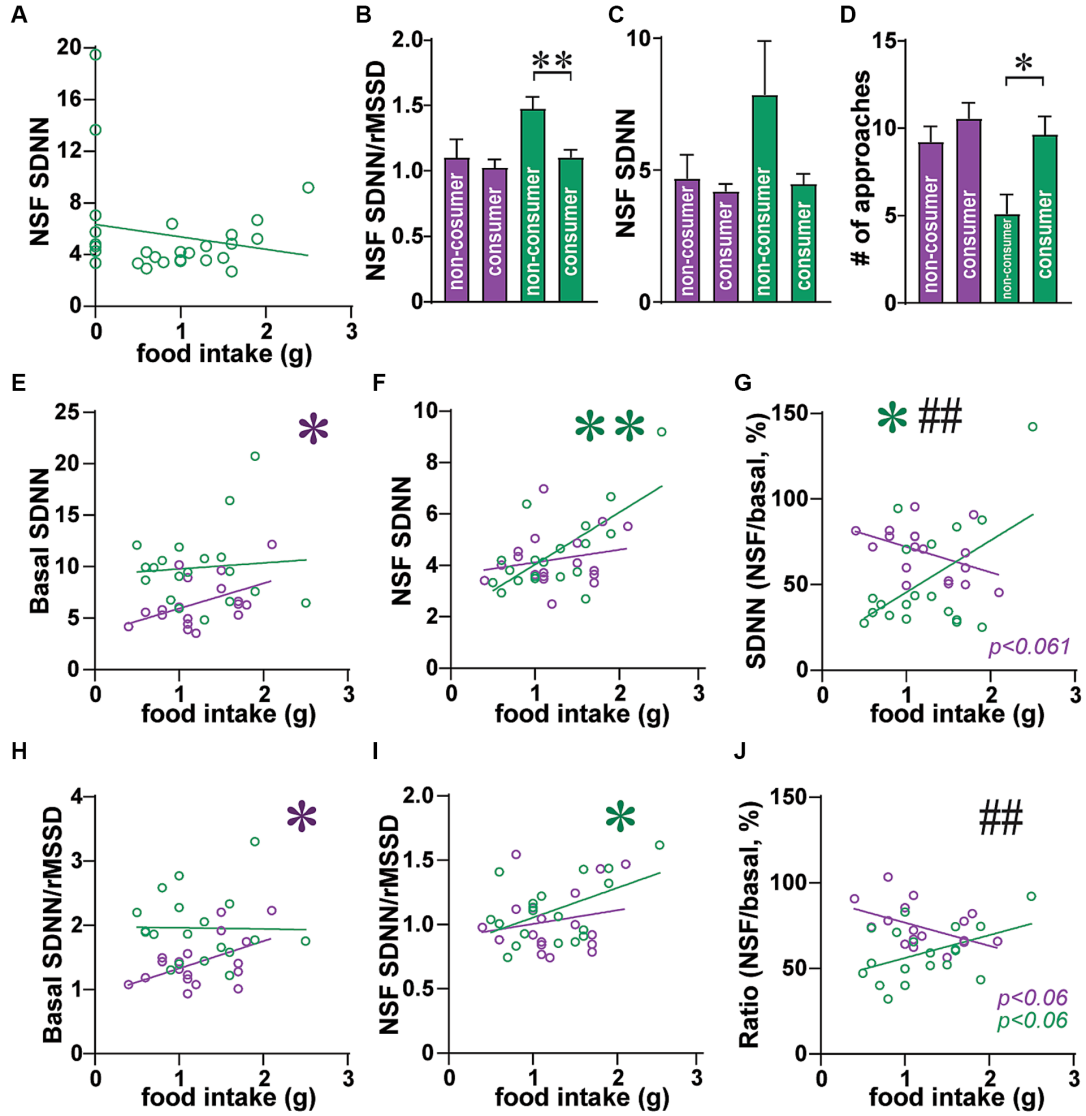
In male consumers, greater SNS indicators predicted higher food intake. **(A)** Same data as Figure 3F but only males, with non-consumers at 0 g food intake, and consumers above that. **(B–D)** Male non-consumers had **(B)** greater SDNN/rMSSD, **(C)** trend for greater SDNN, and **(D)** fewer approaches, than males that consumed food during NSF. **(E–J)** data are for rats that consumed >0 g food during NSF. **(E)** Greater basal SDNN in females, but not males, was correlated with higher intake. **(F)** Greater SDNN during NSF predicted higher food intake in males but not females. **(G)** For the drop in SDNN (from basal to NSF), smaller drop in males related to higher intake, with the opposite trend in females (bigger SDNN drop for more intake). **(H–J)** SDNN/rMSSD showed a similar pattern as SDNN, with **(H)** higher basal ratio relating to greater female intake, **(I)** greater ratio during NSF associated with more male intake, and **(J)** opposite trends in females and males [similar to **(G)**]. *,** *p <* 0.05, *p <* 0.01. ## *p <* 0.01 female–male difference in slopes.

**Figure 9:**
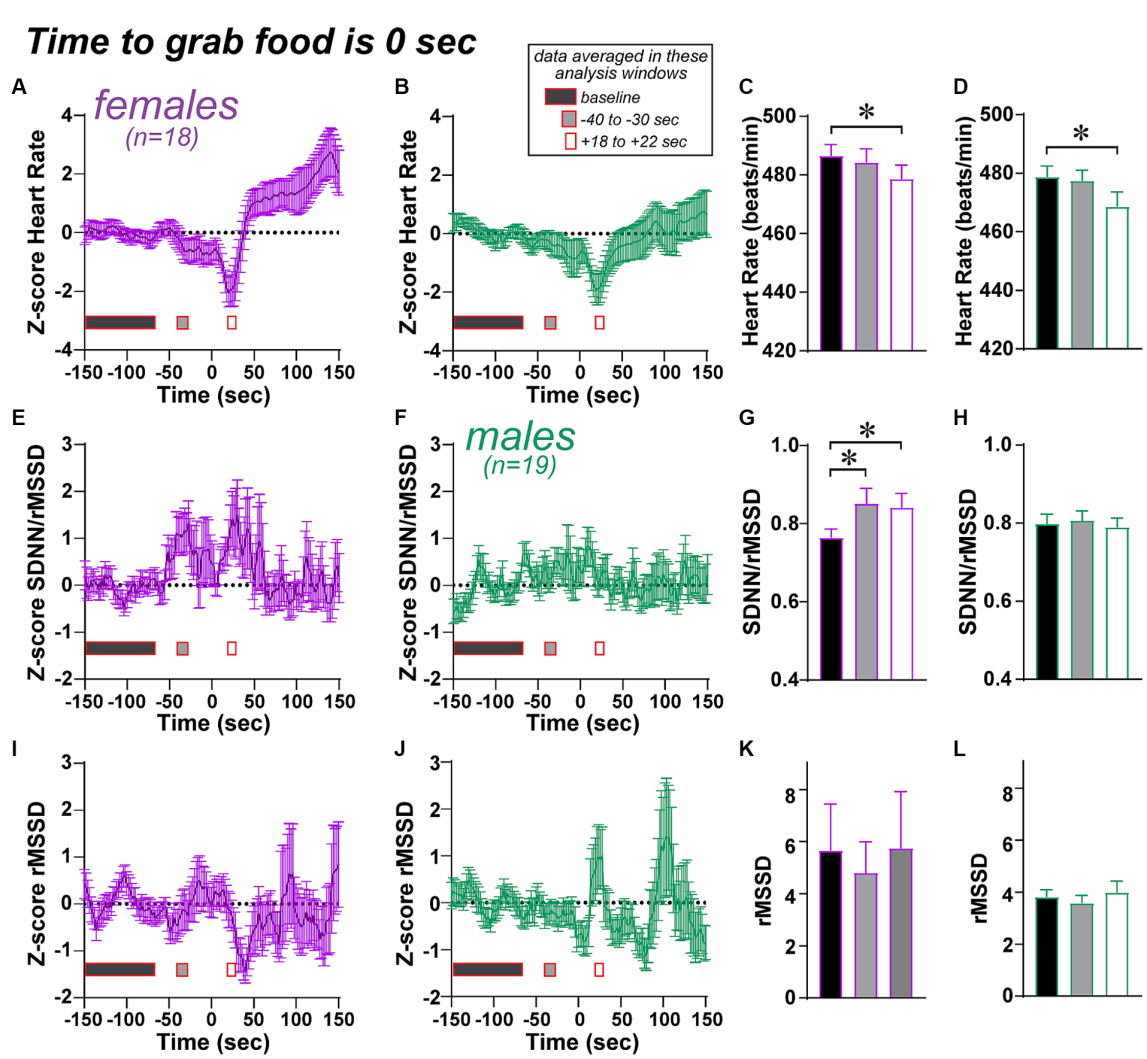
Limited changes in HR/HRV measures across time, centered on the time to grab food, an important step in the NSF. See Methods for how the by-time data were generated. **(A–D)** Both females and males show a dip in heart rate after the food grab. Note that, for a one-way ANOVA, data were first normalized by *Z*-score to a baseline period (150 to 70 s before grab food), then compared across baseline, an interval 40 to 30 s before food grab (gray rectangles in figure), and an interval 18 to 22 s after food grab (white rectangles, about the peak of the heart rate drop after food grab). **(E–H)** Females showed an increase in SDNN/rMSSD both before and after the food grab, while males did not. **(I–L)** No changes in SDNN before food grab. * *p* < 0.05.

For studies in Figures 2, 4–8, we assessed HRV across the whole NSF period, and related HRV measures to overall behavior measures. It would be useful to assess autonomic changes related to specific moments during the NSF task. For these analyses (shown in Figure 9), we ran a 16-s sliding window program (in MATLAB) along the NSF session in which we analyzed average heart rate and the different HRV measures. For example, we first calculate the HR/HRV values centered around 8 s into the task (averaging across data from 0 to 16 s in a given session). This window was then advanced in 2 s steps, and a new average was computed (e.g., where the next interval examined was from 2 to 18 s in the session). In this way, we could assess the HRV from 8 s before to 8 s after time points across the NSF task. We then took this by-time data and centered the results from each session on the time to grab food, and examined HR/HRV measures from 150 s before grabbing food to 150 s after. In addition, we *Z*-scored each HR/HRV measure in a given session relative to the average and standard deviation of data from 150 to 70 s before the time to grab food. Across all rats, there were limited changes in HR/HRV measures before the time to grab.

Analysis of longer periods of heart beats allows more precise and accurate HRV assessment. However, a 5 min period is often utilized for a shorter baseline in humans (64). In addition, recent work has suggested that shorter analysis windows give meaningful HRV patterns, and 60s analysis windows are often as good as a 5 min window for assessing SDNN, RMSSD, and HR (64, 65). Rodent studies have also utilized 1 min HRV analysis windows (66), including for anxiety-like tests (61). However, rat heartbeat in rats is ~4 times faster than human, and so 16 s of rat telemetry will have about the same number of heart beats as 60 s in humans. Thus, we chose 16 s as a sliding window.

For one female, video tape was lost, so we only had food consumed in NSF. In a second female, video stopped early, so we only had latency to approach and food consumed. Also, we did not adjust food intake during NSF for body weight, which is consistent with other work (57, 58). Finally, as an additional comparison, we examined food intake in a 30 min session home cage session after NSF testing.

Since our rats were food restricted for three days before NSF testing, it is possible that food restriction itself altered HR/HRV measures. We had 23 female and 21 male rats with recording during normal food conditions (10 min long), which we then compared, within-rat, to the HR/HRV measures during the 10 min baseline before NSF testing. Relative to the non-food-restricted condition, rats under food restriction had ~5% lower heart rate in males (*t*_(20)_=3.720, *p =* 0.0014) and ~ 2% lower in females (*t*_(22)_=1.616, *p =* 0.1204 paired t-test), with a trend for sex difference in the heart rate change (MW *p =* 0.0534). In addition, SDNN under restriction was ~42% greater than non-restriction in males (*W* = 159, *p =* 0.0043 Wilcoxon ranked sum), but only ~8% greater in females (*W* = 92, *p =* 0.1695), and with a sex difference in SDNN change (MW *p =* 0.0202). In contrast, rMSSD increased with food restriction in both sexes vs. non-restricted condition, with ~27% greater rMSSD in males (*W* = 183, *p =* 0.0007) and ~ 16% increase in females (*W* = 204, *p =* 0.0011), and no sex difference in rMSSD change (MW *p =* 0.1902). Finally, restriction was associated with non-significant changes in SDNN/rMSSD (males: ~10% increase in restricted conditions, *W* = 99, *p =* 0.088; females: ~5% decrease, *W* = −54, *p =* 0.4274), although there was a significant sex difference in ratio change when restricted vs. non-restricted (MW *p =* 0.0230). Thus, food restriction itself did have effects on HR/HRV measures. However, we note that (1) females had basal HR/HRV differences from males that mirror those seen in human and other rat studies, (2) the suggested female use of PNS, and male use of SNS, for eating food during NSF parallels sex differences in autonomic utilization in humans, (3) our previous work suggests the initiation of a task, and highly life-relevant aspects of a task (food in the NSF), are particularly important for females, and here we observe the female HR/HRV predicts the first approach and food eaten, but not more intermediate behaviors (like time in center) that are linked to autonomics in males, and (4) parallel studies of alcohol drinking when not food deprived (submitted in a separate manuscript) found that basal HR/HRV state critically regulated drinking in females but not males, and here we observed that female but not male basal autonomic state was brought into and impacted NSF behavior. Thus, even though there are some sex differences in HR/HRV changes related to food restriction, it is likely that our findings reflect meaningful sex differences in autonomic recruitment under different stages of challenge.

### Statistics

Analyses were performed using two-way repeated measures ANOVA (2way-RM ANOVA), unpaired or paired *t*-tests, *F* test (for differences in variance) or Pearson’s correlations, where appropriate, using GraphPad Prism. For 2way-RM ANOVA comparing basal versus NSF measures (within-subject), and across females and males, we log-normalized the data for SDNN, rMSSD, and SDNN/rMSSD, as often done for HRV (31). For correlations, we showed the raw HRV data, and very similar overall patterns were seen when using log-normal data (Supplementary Figures S10–S13). Results shown are mean ± standard error of the mean.

For almost all conditions that did not show significance, the statistical information is described in the figure legends.

## Results

### Behavioral NSF measures

The Novelty Suppression of Feeding (NSF) task was designed to assess conflict between drive for food and aversion to a bright light in a food-restricted rat, with food and light at the center of the NSF arena (Figure 1A). The NSF task involves a 10 min session where a rat can execute a series of behavioral measures including (1) latency to first approach the center, (2) number of approaches to center, (3) total time spent in center (tolerance of the bright light), and (4) latency to first grab food, all of which progress toward the main goal of the task, which is ultimately (5) food intake (illustrated in Figure 1A). While the main measure of interest is typically food consumed, other measures also provide valuable insight into processing of different aspects of anxiety-like responding (see Introduction). Overall, there were no significant average sex differences between any of the NSF-related behaviors, including food intake (Figure 1B, Mann–Whitney, MW = 276, *p =* 0.5037), latency to first approach the center (Figure 1C, MW = 297, *p =* 0.9999), number of approaches (Figure 1D, MW = 193, *p =* 0.0593), time in center (Figure 1E, MW = 240, *p =* 0.3719), or latency to first grab food (Figure 1F, MW = 260, *p =* 0.6225). However, there were significant sex differences in variance for latency to first approach (*F*_(1, 26)_=15.34, *p <* 0.0001) and time in center (*F*_(1, 26)_=24.46, *p =* 0.0333) (while other behavior measures were not significant, *p* > 0.1). As we will see, latency to approach, which was more variable in females (Figure 1C), related to HRV only in females. In contrast, time in center, which was more variable in males (Figure 1E), was associated with HRV only in males.

### HR and HRV measures: basal and overall changes with NSF testing

Figure 2A shows examples of heart rate traces, and Figure 3 summarizes how the different HR/HRV measures are determined and interpreted. Female and male rats had significant basal differences in HR and HRV, with greater HR and lower SDNN in females, but no rMSSD differences, similar to patterns seen in humans (31) and other rat studies (42). For HR (Figure 2B), there was a significant effect of sex (*F*_(1, 48)_ = 12.15, *p =* 0.0011), basal versus NSF (*F*_(1, 48)_ = 59.35, *p <* 0.0001), and interaction (*F*_(1, 48)_ = 13.17, *p =* 0.0007) (2way-RM ANOVA). Thus, female basal HR was higher than males, and males had a greater rise in HR than females between baseline and NSF. For SDNN (Figure 2C), there was a significant effect of sex (*F*_(1, 48)_ = 13.81, *p =* 0.0005), basal versus NSF (*F*_(1, 48)_ = 84.48, *p <* 0.0001), and interaction (*F*_(1, `48)_ = 10.68, *p =* 0.0020). Thus, female basal SDNN was lower than males, and males had a greater drop in SDNN than females. For rMSSD (Figure 2D), there was a significant effect of baseline versus NSF (*F*_(1, 48)_ = 7.903, *p =* 0.0071) but not sex (*F*_(1, 48)_ = 1.054, *p =* 0.3097) or interaction (*F*_(1, 48)_ = 2.396, *p =* 0.1282), suggesting only minor changes in rMSSD related to a small decrease within the NSF task in males. Further, SDNN/rMSSD (Figure 2E) showed a significant effect of sex (*F*_(1, 48)_ = 19.30, *p <* 0.0001), basal versus NSF (*F*_(1, 48)_ = 139.3, *p <* 0.0001), and interaction (*F*_(1, 48)_ = 12.23, *p =* 0.0010). Scatterplots of these data are shown in Supplementary Figure 3. Similar patterns were observed when calculating percent change in each measure (Figures 2F–I), with significant sex differences for HR (Figure 2F) (MW = 183, *p =* 0.0125), SDNN (Figure 2G) (MW = 164, *p =* 0.0038), and SDNN/rMSSD (Figure 2H) (MW = 154, *p =* 0.0019) but not rMSSD (Figure 2I) (MW = 232, *p =* 0.1298). Finally, post-test intake was significantly greater in males, and did not correlate with food intake during the NSF test in either sex (Figure 4). In sum, males had lower HR, greater SDNN and greater SDNN/rMSSD than females at baseline and showed larger changes in these measures within the NSF task.

### HRV measures and food intake

We first examined how HR and HRV measures might relate to food intake across all rats either from baseline or from within the NSF task. We found that female–but not male–rats overall had HRV measures that predicted food intake. HR alone did not predict food intake, at baseline (Figure 5A), during NSF (Figure 5B), or as a percent change from NSF to baseline (Figure 5C). In contrast, SDNN was significantly related to food intake in females but not in males. More specifically, in females, the relationship between food intake and baseline SDNN was almost significant (Figure 5D; *F*_(1, 21)_=4.232, *R*^2^ = 0.168, *p =* 0.0523), but not for NSF SDNN (Figure 5E; *F*_(1, 21)_ = 0.021, *R*^2^ = 0.001, *p =* 0.8850). However, females with a greater drop in SDNN from baseline to NSF (expressed as percent) had significantly greater intake (Figure 5F; *F*_(1, 21)_ = 8.672, *R*^2^ = 0.292, *p =* 0.0077). In males, food intake did not relate to any SDNN measure (Figures 5D–F).

SDNN relates to both SNS and PNS tone, whereas rMSSD only indexes PNS influence (Figure 3). Thus, any effects through PNS would also impact rMSSD. However, food intake was not correlated with any rMSSD measure (Figures 5G–I). On the other hand, similar to SDNN (Figure 5F), reduced SDNN/rMSSD also predicted greater food intake in females. Food intake did not relate to SDNN/rMSSD at baseline (Figure 5J) or during NSF (Figure 5K). However, a larger decrease in SDNN/rMSSD from baseline to NSF (expressed as percent) correlated with significantly greater food intake in females (L; *F*_(1, 21)_ = 6.694, *R*^2^ = 0.242, *p =* 0.0172) but not males (Figure 5L; *F*_(1, 25)_ = 0.233, *R*^2^ = 0.009, *p =* 0.6333), and with a significant difference in slope between the sexes (*F*_(1, 46)_ = 4.775, *p =* 0.0340). Lower SDNN/rMSSD ratio and lower SDNN both suggest reduced SNS and thus greater PNS influence. Thus, these findings are consistent with studies suggesting greater PNS utilization in women and SNS in men.

### HRV measures and latency to first approach the center

While food intake is an important measure in NSF, the task is only 10 min, and thus behaviors more distal from actual food contact are also of interest. Thus, we next examined the latency to first approach the center. Interestingly, females showed a strong relation between several HRV measures and the latency to first approach the center, which were not observed in males. In addition, for several measures, the baseline HR/HRV level predicted subsequent latency to approach during the NSF, suggesting that the basal autonomic state of females prior to NSF continued during NSF testing and impacted behavior. In particular, in females, longer latency to approach was related to significantly higher HR at baseline (Figure 6A; *F*_(1, 20)_ = 5.933, *R*^2^ = 0.229, *p =* 0.0243) and during NSF (Figure 6B; *F*_(1, 20)_ = 14.18, *R*^2^ = 0.415, *p =* 0.0012), but not in males (Figures 6A,B). Female SDNN at baseline did not correlate with approach latency (Figure 6C; *F*_(1, 20)_=2.341, *R*^2^ = 0.105, *p =* 0.1417), while reduced SDNN during NSF did (Figure 6D; *F*_(1, 20)_=11.43, *R*^2^ = 0.364, *p =* 0.0030). Furthermore, lower female rMSSD at baseline (Figure 6E; *F*_(1, 20)_=5.933, *R*^2^ = 0.229, *p =* 0.0243) and during NSF (Figure 6F; *F*_(1, 20)_=4.575, *R*^2^ = 0.186, *p =* 0.0450) both correlated with longer latency to first approach. However, in males, no HRV measures correlated with latency to first approach (Figures 6C–F). Finally, with significant effects for both rMSSD and SDNN in females, latency to first approach was not correlated with SDNN/rMSSD (Supplementary Figure 4). Thus, these results suggest that basal autonomic state in females persisted into NSF testing, such that females with higher HR and reduced PNS and SNS (but no shift in SNS-PNS balance) were delayed in their first approach to center.

One consideration is that higher HR could reduce potential for variability. However, even though HR overall did correlate with lower HRV, there was some dynamic range (Supplementary Figure 5). Lastly, female HR did not correlate with several other behavioral measures.

### HRV measures and number of approaches and time in center

Unlike females, no HRV measure predicted latency to first approach the center in males (Figure 6). In strong contrast, several HRV indicators did predict male but not female NSF behaviors that were intermediate between the first approach and food intake, in particular the number of approaches to center and time spent in center. However, these measures did not relate to HR at basal or during NSF (Supplementary Figure 6). Also, basal SDNN was not related to the number of approaches (Figure 7A) or time in center, although with a trend in males (Figure 7C; males: *F*_(1, 25)_ = 3.640, *R*^2^ = 0.128, *p =* 0.0680). In contrast, lower SDNN during NSF was correlated with significantly more approaches in males (Figure 7B; *F*_(1, 25)_ = 4.634, *R*^2^ = 0.156, *p =* 0.0412) but not in females (Figure 7B; *F*_(1, 19)_ = 1.192, *R*^2^ = 0.059, *p =* 0.2886). Lower NSF SDNN also correlated with significantly greater time in center in males (Figure 7D; *F*_(1, 25)_ = 4.860, *R*^2^ = 0.163, *p =* 0.0369) but not females (Figure 7D; *F*_(1, 19)_ = 0.035, *R*^2^ = 0.002, *p =* 0.8531), and the slope for NSF SDNN was significantly different between the sexes (*F*_(1, 44)_=4.921, *p =* 0.0317). However, rMSSD did not relate to number of approaches at baseline (Figure 7E) or during NSF (Figure 7F), or to time in center at baseline or during NSF (Figures 7G,H).

With reduced SDNN but not rMSSD related to greater male engagement with the center of the NSF arena, one might predict that lower SDNN/rMSSD would also predict higher male behavior. However, basal ratio did not predict number of approaches (Figure 7I) or time in center (Figure 7J), although there was a trend in males (Figure 7K; males: *F*_(1, 25)_ = 3.813, *R*^2^ = 0.132, *p =* 0.0622). In strong contrast, males with lower SDNN/rMSSD during the NSF had significantly more approaches (Figure 7J; *F*_(1, 25)_ = 19.34, *R*^2^ = 0.436, *p =* 0.0002) and time in center (Figure 7L; *F*_(1,25)_ = 8.727, *R*^2^ = 0.259, *p =* 0.0067). This was not seen in females (Figure 7J; number of approaches: *F*_(1, 19)_ = 0.365, *R*^2^ = 0.019, *p =* 0.5529; Figure 7L; center time: *F*_(1, 19)_ = 0.403, *R*^2^ = 0.021, *p =* 0.5330). Furthermore, there was a significant sex difference in slope of SDNN/rMSSD for time in center (*F*_(1, 44)_ = 7.576, *p =* 0.0086). Similarly, males with a larger change in SDNN/rMSSD from baseline to NSF also had more approaches (Supplementary Figure 7). Taken together, these findings suggest that males with lower SNS during the NSF (lower SDNN and ratio, but no differences in rMSSD) made significantly more approaches and spent more time in the center (tolerating the light). Females did not display these patterns, suggesting that reduced SNS could promote different aspects of behavior in females (first approach, food intake) and males (number of approaches, time in center).

### Exploratory analysis: potential differences between consuming and non-consuming rats

When examining Figure 1B, we noted that a set of rats ate nothing in the NSF task and that these were somewhat segregated, as a population, from rats that ate some amount of food. Indeed, the distribution of male food intake was not normal when including non-consumers (Shapiro–Wilk = 0.9177, *p =* 0.0348 not normal), but was normal when only including consumers (Shapiro–Wilk = 0.9773, *p =* 0.8820, therefore normal). Since rats only had a 10 min NSF period, restricting the time allowed for intake, we examined how HRV measures might relate to NSF behaviors only in rats that consumed some food during the NSF. Indeed, as shown in Figure 8A (similar to Figure 5E), the relation between male food intake and SDNN during the NSF could have been U-shaped, with higher SDNN in non-consumers, lower SDNN in lower-intake consumers, and somewhat higher in higher-intake consumers. For example, male non-consumers had higher SDNN/rMSSD ratio (Figure 8B; MW = 21 *p =* 0.0024) and a trend for higher SDNN during NSF (Figure 8C, MW = 40, *p =* 0.0583), compared to male consumers, as well as fewer approaches than consumers (Figure 8D; MW = 31, *p =* 0.0148). This subpopulation of non-consuming rats could reflect a different profile of autonomic activation, and thus we sought to better understand how food eaten related to HR/HRV among the majority of rats that did consume some food.

After removing non-consumers, higher basal SDNN related to greater subsequent intake in females but not males (Figure 8E; females: *F*_(1, 16)_=4.536, *R*^2^ = 0.221, *p =* 0.0491; males: *F*_(1, 17)_=0.117, *R*^2^ = 0.006, *p =* 0.7361). In strong contrast, males with higher SDNN during NSF had higher food intake (Figure 8F; *F*_(1, 17)_ = 15.05, *R*^2^ = 0.470, *p =* 0.0012), while females did not (Figure 8F; *F*_(1, 16)_ = 0.738, *R*^2^ = 0.044, *p =* 0.4029). Further, for males, a smaller drop (expressed as percent) in SDNN from basal to NSF predicted greater intake (Figure 8G; *F*_(1,17)_ = 6.103, *R*^2^ = 0.264, *p =* 0.0244), with the opposite pattern in females (*F*_(1, 16)_ = 4.066, *R*^2^ = 0.203, *p =* 0.0609). This trend in females was similar to that in Figure 5F, and there was a significant difference in slopes between females and males (*F*_(1, 33)_=8.732, *p =* 0.0057). These results suggest that, in rats that consumed some food, greater food intake in males was associated with higher SNS during NSF to a point, after which too high an SNS activation resulted in no food consumption in males. In females, higher intake related to greater SNS at baseline, such that a larger SDNN drop in females from baseline to NSF predicted higher consumption.

If this interpretation were correct, we would expect that SDNN/rMSSD during NSF would predict food intake in males, while SDNN/rMSSD at baseline would predict amount eaten in females. In agreement, higher basal SDNN/rMSSD predicted greater intake in females but not males (Figure 8H; females: *F*_(1, 16)_ = 5.561, *R*^2^ = 0.258, *p =* 0.0314; males: *F*_(1, 17)_ = 0.006, *R*^2^ = 0.000, *p =* 0.9394). Conversely, higher SDNN/rMSSD during NSF predicted greater intake in males (Figure 8I; *F*_(1, 17)_ = 5.790, *R*^2^ = 0.254, *p =* 0.0278) but not females (Figure 8I; *F*_(1, 16)_ = 0.639, *R*^2^ = 0.038, *p =* 0.4359). When examining the drop in SDNN/rMSSD (Figure 8H), males had a trend for lower drop in ratio correlating with higher intake (*F*_(1, 17)_ = 4.131, *R*^2^ = 0.196, *p =* 0.0580) and females a trend for higher ratio drop related to greater intake (*F*_(1, 16)_ = 4.333, *R*^2^ = 0.213, *p =* 0.0538). However, the female and male slopes were significantly different (*F*_(1, 33)_ = 8.120, *p =* 0.0075). Thus, these findings agree with the possibility that higher SNS during NSF increased intake in male consumers, while higher baseline SNS in females, and a larger SNS drop when moving to NSF, were associated with greater female intake.

### Relationship across NSF behavioral measures

Since we observed sex differences in specific NSF behaviors that related to HRV measures, it was also useful to understand how different NSF behavioral measures predicted each other. While HRV indicators were associated with female latency to first approach, and male number of approaches, neither of these behavioral measures correlated with food intake (Supplementary Figures 8A,B). In contrast, latency to grab food significantly correlated with food intake, where rats that grabbed earlier ate more food (Supplementary Figure 8C). However, latency to grab food did not relate to any HR/HRV measure (not shown). While the interpretation remains uncertain, it may relate to the briefness of the food-grabbing activity, and rats often took food back to the periphery to eat. Further studies would be required to better understand these possibilities.

### Analysis of HR/HRV measures across time in the session, centered on the time to grab food

One question is whether there were changes in any cardiac measures across the NSF session. To determine this, we averaged HR/HRV measures using a 16-s sliding window across the session. We then centered this data around time to grab food, which was time 0, and extending from 150 s before food grab to 150 s after (see Figure 9 and Methods). To assess HRV changes in the time before grab food, we generated a baseline (averaged across 150 s to 70s before the time to grab food), and all data values were *Z*-scored relative to this average and standard deviation. This was done separately for each HR/HRV measure for each session. In this way, we could visualize potential changes across the pre-food grab and post-food grab periods. Figure 9 shows such plots for HR Figures 9A,B, SDNN/rMSSD Figures 9E,F, and SDNN Figures 9I,J; rMSSD is shown in Supplementary Figure 9.

To analyze these data, we noted visually that some periods of time might show significant differences from baseline, especially before and after grab in females for SDNN/rMSSD, and after food grab for HR in both sexes. With the great number of possible post-hoc comparisons (for an ANOVA with so many time points), we chose to examine possible HR/HRV changes in two analyses windows, one 30–40 s before the grab (“-30s” grey rectangles; where females seemed to show increased SDNN/rMSSD), and the other ~20 s after the grab (“+20s” white rectangles; from 18 to 22 s after grab food, around the peak of the HR drop). Indeed, both sexes showed significantly lower HR in the +20-s analysis window versus baseline (Figures 9C,D; 1-way repeated measures ANOVA: females, *F*_(2, 18)_ = 4,163, *p =* 0.0249; males, *F*_(2, 18)_ = 8.400, *p =* 0.0018; post-hoc females *p =* 0.0202, males *p =* 0.0120). For the other HRV measures we used a Friedman test with Dunn’s post-hoc as a non-parametric variant of 1-way repeated measures ANOVA. The SDNN/rMSSD measure in females was elevated at both the −30s and + 20s analysis windows (Figure 9G, Friedman stat = 10.33, *p =* 0.0057, basal vs. −30s *p =* 0.0373, basal vs. +20s *p =* 0.0081), with no relation in males (Figure 9H, Friedman stat = 0.4211, *p =* 0.8102). In addition, there were no differences across analysis time points for SDNN (Figures 9K,L, female: Friedman stat = 1.444, *p =* 0.4857, male: Friedman stat = 4.526, *p =* 0.1040) or rMSSD (Supplementary Figure 9). The similar statistics for SDNN and rMSSD in females in this rank-sum measure raises the speculation that SDNN and rMSSD are more tightly coupled in females than males. Also, after the food grab and the transient HR dip, HR levels were significantly greater in females (*t*_(17)_ = 2.234, *p* = 0.0392, paired *t*-test), which was not observed in males (*t*_(18)_ = 0.011, *p* = 0.9911).

One final question is whether the greater SDNN/rMSSD in females before food grab relates the time to grab or food eaten measures. However, the increase in SDNN/rMSSD did not correlate with time to grab food (*F*_(1, 16)_ = 0.094, *p* = 0.7631) or with food eaten (*F*_(1, 16)_ = 0.121, *p* = 0.7326). Taken together, these findings suggest the possibility that HR/HRV measures were, on average, relatively consistent before the food grab, with some periods of change in some measures. As such, there was greater SDNN/rMSSD in females before and after food grab, which could suggest a sex-specific HRV regulator of food grabbing in the NSF, as well as a small but significant decrease in HR after grabbing food in both sexes.

## Discussion

**Figure 10:**
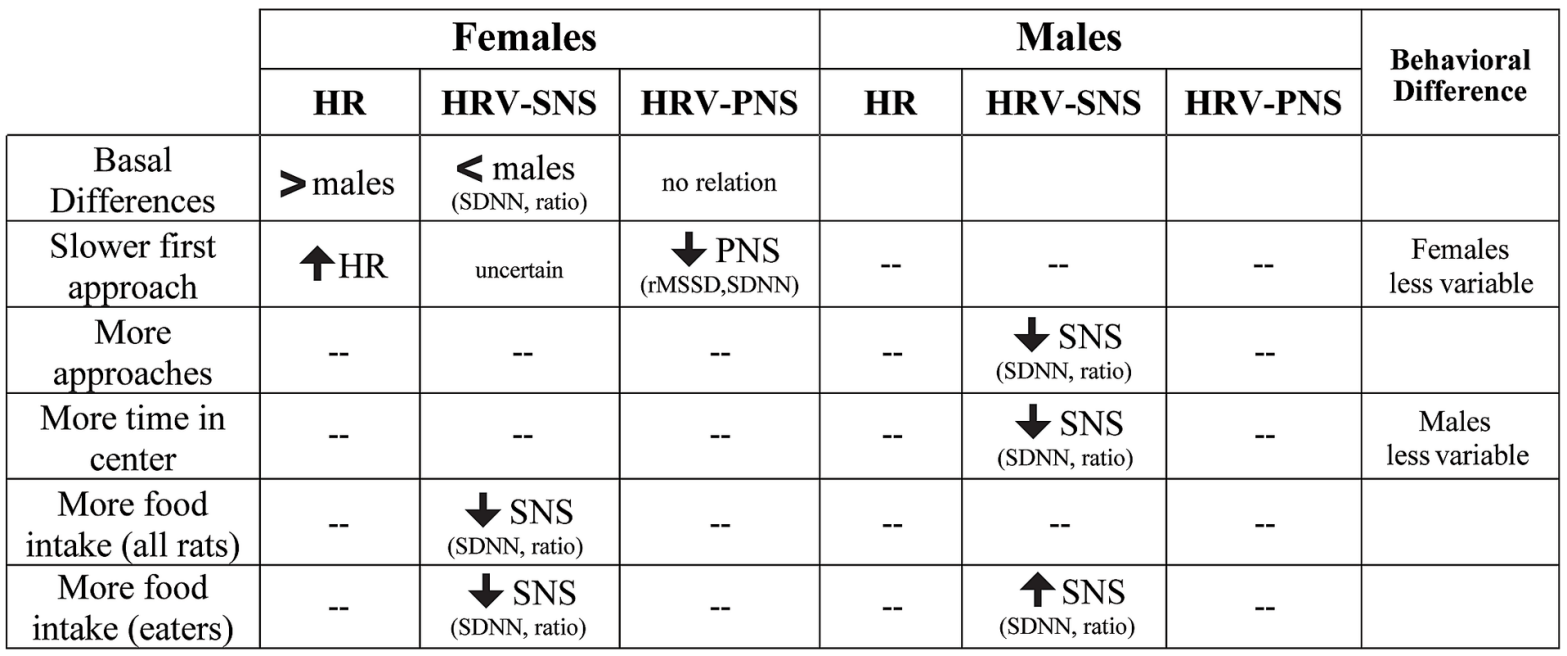
Summary of sex differences in the relation between HRV patterns and specific NSF behaviors. For females, greater HR at baseline and during NSF predicted slower initial latency to approach, while lower SNS correlated with greater food intake. In males, lower SNS was associated with increased number of approaches and time in center, while greater SNS predicted higher food intake in rats that consumed food. “--” indicates no relation between HRV and behavioral measures.

Women have nearly twice the prevalence of anxiety disorders, but critical mechanisms underlying sex differences in expression of anxiety remain incompletely understood. We utilized cardiac telemetry to collect data on HR and HRV, since these have demonstrated potential as important biomarkers for and contributors to pathological states, including neuropsychiatric conditions. In addition, HR/HRV measures could help to better understand sex differences in anxiety-related autonomic mechanisms. Thus, we examined cardiac telemetry in food-restricted adult rats during the novelty suppression of feeding (NSF) task, which presents rats with a conflict between food and light at the center of the NSF arena, with results summarized in Figure 10. In females, basal HR was greater than in males, and basal SNS-related indicators (SDNN and SDNN/rMSSD) were lower than in males. While apparently paradoxical to have a higher resting HR in addition to a higher SDNN and SDNN/rMSSD ratio, these patterns are similar to what is seen in a large recent human study (31), and suggest that rat telemetry is robustly assessing HR/HRV function. While the precise reasoning for this apparent paradox is not yet fully understood, the same large human study suggests the sexes are perhaps different in their sensitivities to acetylcholine on cardiac chronotropy, which may be an effect of estrogen (31). Furthermore, in the NSF, females (but not males) with higher basal HR and lower PNS index (reduced rMSSD) were slower to make the first approach to center, suggesting sex differences in basal autonomic state that females brought into and impacted initial NSF behaviors. In contrast, males (but not females) had HRV patterns suggesting that reduced SNS function during NSF (lower SDNN and SDNN/rMSSD, without changes in rMSSD) led to an increased number of approaches to center and time in center. Finally, across all rats, females with lower SNS indicators consumed significantly more food, with no overall relation in males. However, there were two potentially distinct subpopulations of rats, some that consumed no food during the 10 min NSF, and most rats that consumed some food. Non-consuming males had significantly higher SNS measures (higher SDNN/rMSSD) and reduced approaches than male consumers. Importantly, among consumers, males with greater SNS indicators ate more food up to a point, after which we posit SNS perhaps became active enough to inhibit food intake in the non-consuming males. This is the opposite to what we observed in females (lower SDNN and SDNN/rMSSD), with no differences in autonomic indices related to whether food was consumed within the task. Interestingly, this is congruent with human findings suggesting that women engage PNS more, and men SNS more, during autonomic regulation under challenge. Thus, our findings show novel and likely clinically relevant sex differences in HRV-behavior relationships. In addition, our results suggest that by examining HRV measures during different aspects of anxiety-like responding within the NSF task (baseline, initial approach to center, overall approaches to center, time to grab food, and food intake), we may gain new insights into sex-specific vulnerabilities and expression of anxiety-like behavior.

Many groups have suggested that women engage the PNS more during autonomic regulation under challenge, while men engage the SNS more (15, 26, 34–36), and such findings align with sex differences appreciated in HRV brain circuitry (discussed below). Our findings are, to our knowledge, the first to provide clear functional implications of the PNS-SNS sex differences using HRV indices, and, interestingly, that these HRV measures related to different aspects of anxiety-like behavior within the NSF task. Our findings might suggest that females with higher basal SNS (higher HR) and lower basal PNS (lower rMSSD) brought this autonomic state into the beginning of the NSF task, where such autonomic engagement significantly slowed the latency to first approach the center. However, females likely then disengaged from this state, since lower SNS indices were associated with greater food intake, where reduced SNS was indicated by lower SDNN and SDNN/rMSSD without changes in rMSSD, suggesting that SNS influence decreased without PNS alterations. In contrast, basal HR/HRV measures in males did not relate to any NSF behavior. Instead, males with lower SNS during NSF (reduced SDNN and SDNN/rMSSD with no significant change in rMSSD) had a greater engagement with the task center, shown through more approaches to and greater time spent in the brightly lit arena center. However, males also likely had a shift in balance, since exploratory analyses suggested that males with greater SNS either ate no food at all (non-consumers) or, for males that did consume some food, higher SNS related to greater intake level. Thus, we posit that perhaps males that engage SNS up to a point are able to consume food in the novel context, but after such point the SNS engagement is possibly overwhelming and leads to no food consumption. Such findings could be used to improve and individualize anxiety treatment, not merely by sex but also by individualized understanding of one’s unique autonomic engagement. One such example for women would be that the different HRV changes at baseline, early in anxiety testing, and for different anxiety measures, could all come together to provide a robust constellation of biomarkers for those with particular risk for and/or expression of anxiety states.

We also note that, more generally, female rats had higher HR and lower SDNN, with no differences in rMSSD. These patterns were similar to what has been reported in humans (31) and rats (42). The presence of these basal differences suggests that our telemetry was accurately and robustly assessing HR and HRV parameters and validates that other HRV differences we observed are likely to be translationally and clinically relevant. However, we also note the underlying mechanisms for the apparent paradox in female autonomics, with great HR and also HRV, remain unclear, several studies have posited that sex differences in cardiac acetylcholine regulation could contribute to this apparent paradox (31, 35).

It is also interesting that female HRV measures were particularly related to the first challenge-related movement in the task (the latency to first approach the center, and also food intake). In this regard, our previous work suggests that female anxiety-like behavior is only greater than males for aspects of a task that might be considered most life-relevant, including food intake in NSF and the first movement in the novel context (8) (as mentioned in the Introduction). We note that our present studies did not observe sex differences in the level of these NSF behaviors, unlike the previous study (8). Several factors may contribute to this, including use of a larger NSF arena here [100 × 100cm here, 60 × 60cm in (8)], and where rats in the present work had undergone telemetry surgery ~10 days before NSF testing, both of these factors could plausibly increase stress or anxiety in the rat. Nonetheless, even though we did not observe behavioral differences here, the relation between female HRV and first latency to approach the food as well as food intake concurs conceptually with our previous work. In particular, we had compared female and male rat behavior in NSF and in the Light–Dark Box (LDB), which we consider a control task since it involves aversion to bright light, but no food restriction (8). Importantly, we used a moderate dose of the anxiolytic diazepam (67, 68) to assess whether any sex differences actually reflect anxiety (e.g., vs. locomotion). Interestingly, only a subset of anxiety measures show sex differences. In NSF, these are measures directly related to food (latency to grab food, amount of food eaten), where females show greater anxiety, and diazepam causes more anxiolysis in females. In strong contrast, measures less directly related to food (such as time in the center) are similar in females and males. In addition, LDB responding is largely comparable in females and males, except for the first movement in the task, moving from the lit chamber into the dark (a novel context), where females had more anxiety-like behavior (8).

Taken together, we have interpreted these findings to indicate that females are more impacted by aspects of an anxiety-like task that could be considered more life-threatening or life-relevant, with largely similar anxiety-like expression in females and males for behaviors more distal to the central focus (e.g., grabbing food). Interestingly, results from other studies add additional support for this possibility. When a shocker is placed in the home cage bedding, females bury the shock probe more quickly than males, and diazepam slows latency-to-bury in females more than males; however, there are no sex differences in other behaviors more distal to direct interaction with the shocker (69). Females also have greater anxiety and bigger effect of anxiolytics with predator odor (68, 70). Thus, we hypothesize that females are specifically impacted by life-threatening conditions (food when food restricted; initial uncertainty in a novel context; direct shock; imminent danger). In the present study, we did not observe overt behavioral differences in the first approach to center or for food intake. However, the initial approach in females was strongly delayed when basal HR was higher (and rMSSD lower), while food intake was related to lower SNS indicators (lower SDNN, lower SDNN/rMSSD ratio, no significant change in rMSSD). In contrast, more intermediate behaviors, such as number of approaches and time in center, were not sex-different in the previous study (8), and were not correlated with HRV measures in females, but only in males. One implication is that, even with sex-similar behavior and diazepam sensitivity in the number of approaches and time in center, there were still sex differences in autonomic mechanisms related to these behaviors. Thus, our present HRV studies provide novel insights into underlying mechanisms that would not be detectable with behavioral and even pharmacological methods. Indeed, in some ways, the similar NSF behavior was a strength, since it allowed us to infer that anxiety-like behaviors were related to and likely influenced by different autonomic drivers in females and males.

One key question is whether the greater HR in females, especially during NSF, might reduce HRV simply by mathematically reducing potential for variability, which could confound accurate interpretation of HRV measures. At present, we cannot definitively answer this question. Greater HR did relate to reduced HRV (Supplementary Figure 5), but HR only correlated with one behavioral measure, latency to first approach, and only in females. Especially during NSF, when HR was higher in both males and females, we note that other behaviors (number of approaches, time in center) were related to lower SDNN in males but not in females. Thus, it is unlikely that HR differences explained all the observed sex-related patterns, although we cannot rule out, for example, that the relation between lower rMSDD and slower first approach (Figures 6E,F) is at least in part impacted by greater HR reducing variability.

When our group initially planned these experiments, we had expected to see not only sex differences in anxiety-like responding, but also hypothesized that we would appreciate differences based on alcohol drinking history. This was in part due to results from the human literature that suggest humans with alcohol use disorder have altered basal HRV (13, 41) compared to healthy controls. Furthermore, HRV in alcohol use disorder patients can reliably predict relapse (34). Thus, we had initially hypothesized that rat drinking history would also alter HRV responses, at baseline and during anxiety-like behavior. However, we found no such differences by drinking history, only by biological sex (Supplementary Figures 1, 2). We believe this is due to the model of alcohol drinking we utilize in our rats, which does not recapitulate alcohol dependance but rather facilitates compulsive-like drinking, a critical barrier to treatment in addiction disorders (48, 51) (and see Methods). We choose to model compulsion rather than dependence in part since many problem drinkers are not dependent (71). Thus, the model of alcohol drinking utilized by our lab may not be severe enough to replicate findings from certain human alcohol studies [discussed further in (48, 51)].

Another important caveat of the present study is the use of singly housed animals, since single housing is a stressful stimulus and can affect cardiovascular functioning (72, 73) and anxiety-like behavior [discussed in (8)]. However, part of our reason for single housing was to support the larger goal of understanding interactions between alcohol and anxiety, where single housing is often required for alcohol drinking rat studies. Furthermore, as mentioned above, we had initially hypothesized that alcohol drinking history could affect HR/HRV responses in anxiety-like behavior, and thus planned the study using our typical alcohol drinking protocols which require single housing rats.

It is also worth noting that our rats were food restricted for 3 days prior to testing, and food restriction could plausibly alter cardiovascular measures and increase stress. Indeed, as detailed in the Methods, food restriction did lead to some changes relative to free feeding. However, other groups such as Inagaki and colleagues found that food restriction alone did not alter HRV in rats (74). Thus, the important question of the role of food restriction on HRV remains open, and future work is needed to further clarify these possible effects.

Another important consideration is how autonomic regulation can vary across the menstrual cycle in humans, and estrous cycle in rats. Many studies find clear sex differences in baseline HR and HRV in freely cycling women (15, 31, 34), as we (Figure 2) and others (42) observed in rats. However, estrogen can increase PNS activity in humans (33, 39, 75) and rodents (35). Also, we find clear sex differences in anxiety-like behavior in freely-cycling females versus males (8), where we examined a large sample of females, in different cohorts tested across several days, in a partial, although likely incomplete, attempt to sample across estrous stages. Rodent studies show reduced anxiety-like behavior in proestrus (58, 76, 77), although other groups find no estrous influence in elevated plus maze or open field (9, 78), shocked licking (79), or in light enhancement of startle (80), and ovariectomy alters NSF performance (81). In humans, women exhibit anxiety differences across the menstrual cycle. During aversion responding, there is greater central amygdala (and other regions) activity when estrogen is low versus high, with brain activity under low estrogen more similar to males, suggesting that higher estrogen may reflect an anti-stress mechanism (39, 75, 82, 83). However, one human study found that HFHRV showed greater sex differences (~45%) compared to cycle stage differences (~16%) (36). Thus, some conditions show clear association with estrogen levels, while others may not vary across the menstrual and estrous cycles. Future studies are needed to examine more carefully the relation between estrous cycle and autonomic regulation during anxiety-related conditions.

A final critical question is whether there are sex differences in brain circuits underlying anxiety and autonomic regulation. Many human studies have noted sex differences in expression of affect related behaviors, including neural correlates of emotional reactivity (5, 84–86), e.g., with greater amygdala in women and anterior insula in men in an emotion-evoking task (5). Males also have larger cortisol increases than women during stressors (83). Other work suggests greater top-down regulation of emotion in men versus women (5, 38, 87), perhaps reflecting greater differences in effort and/or cognitive strategies (5, 87). Further, better emotional regulation is related to positive insula-amygdala connectivity in women, with negative connectivity in males (88). Also, greater PNS function is associated with greater amygdala activity in women (but not men) under cognitive challenge (34) and at rest (34, 37), and greater amygdala and insula activation predicts more negative affect during emotion tasks in women but not men (38). As noted above, amygdala activity reflects basal SNS activation by negative images in women during lower estrogen stages of the menstrual cycle (39, 75). In addition, while few studies examine sex differences in HRV circuitry in neuropsychiatric conditions, women with depression (but not men) have higher insula and amygdala activity that relates to lower HFHRV during negative affect (40). Finally, it is likely that brainstem areas (89) also contribute to differences, and sex differences are observed in important arousal systems [noradrenaline (90), orexin (91)] where females do not show habituation to receptor stimulation, leading to protracted sensitivity to stressors. Thus, there are likely important sex differences in brain circuit regulation of anxiety-like behaviors and autonomic regulation. However, there are several challenges when interpreting such studies, including difficulty in disentangling biological versus social influences (2, 5), and where similar behavior may involve different brain areas [e.g., human (92), rodent (93)]. Also, some studies find few sex differences, including in insula activation to stress cues (94, 95). Thus, sex differences in autonomic regulation under challenge, and underlying brain circuits, remain of great and clinically-relevant interest, but are still incompletely understood. Future studies, both in humans and animals, are needed to further disentangle these questions.

Taken together, our studies provide novel and likely clinically-relevant insights into sex differences in autonomic mechanisms that contribute to specific aspects of basal and anxiety-like conditions. Since women have nearly twice the risk of developing an anxiety disorder, and human studies suggest sex differences in use of PNS versus SNS, our results provide important insight into when and how such sex differences manifest before and during anxiety-like behavior.

## Data availability statement

The raw data supporting the conclusions of this article will be made available by the authors, without undue reservation.

## Ethics statement

The animal study was approved by the Institutional Animal Care and Use Committee at Indiana University. The study was conducted in accordance with the local legislation and institutional requirements.

## Author contributions

RF and FH: conceptualization, formal analysis, and writing- original draft preparation. RF, TO, PS, AG, and FH: methodology. RF, TO, and PS: data collection. RF, TO, PS, AG, and FH: data interpretation and methodology. RF, TO, PS, AG, and FH: writing-review and editing. FH: supervision, project administration, and funding acquisition. All authors have read and agreed to the published version of the manuscript.
