# Supplementary material for: Heart rate variability measures indicating sex differences in autonomic regulation during anxiety-like behavior in rats
Source: Front Psychiatry. 2023 Oct 31;14:1244389. doi: 10.3389/fpsyt.2023.1244389 (PMC10644002; doi:10.3389/fpsyt.2023.1244389)

Supplemental Materials

Heart rate variability measures indicating sex differences in autonomic regulation during anxiety-like behavior in rats

Raizel M. Frasier[1,2], Thatiane de Oliveira Sergio[1], Phillip A. Starski[1], Angela J. Grippo[3], F. Woodward Hopf[1,4*]

Suppl.Methods

Alcohol Drinking. Rats first drank under a 2-bottle choice intermittent access to alcohol paradigm, with access to alcohol (20% v/v), or water in a second bottle. Alcohol access began on Monday, Wednesday, and Friday at ~1 hour into the dark cycle, and lasted 16-24 hours each day. Following ~3 months of IA2BC, rats were switched to drink alcohol (20% v/v) or water for 20 min/day Monday-Friday (1-4). Non-drinking rats lived in the same room but never had access to alcohol.

Telemetry Surgery. Alcohol was withheld from rats for approximately 48-72 hours prior to surgery to prevent complications. Using antiseptic surgical techniques, rats were put under isoflurane anesthesia and implanted with a telemetry device (type PTA-M-C, part# E-430001-IMP-130) from TSE Systems Inc. (Chesterfield, MO), with instruction and assistance from TSE personnel. The telemetry device consisted of a silicone elastomer transmitter (8.3 mm in by 16.5 mm x 4 mm), and a thin, plastic-sheathed wire which had a small sleeve at the distal tip which detected changes in blood pressure within the artery. The surgery required two incisions, one in the midline of the abdominal cavity (to place the transmitter), and a second where the abdomen meets the left thigh to access the left femoral artery. The femoral artery was carefully dissected from the adjacent femoral vein and femoral nerve, and then dilated through topical application of 2% injectable lidocaine. Suture silk was used to temporarily occlude the femoral artery, and then a small needle puncture was made into the vessel. The wire with telemeter at the end was inserted into the blood vessel, the silk suture was loosened slightly, and the wire tip advanced until it sat approximately between the iliac bifurcation and the renal arteries within the abdominal aorta. This was assisted by a removable trocar which led the wire. Once the wire was in place, the suture silk was lightly tied to the femoral artery to keep the wire from slipping and to assist in closing the small puncture to prevent bleeding. To confirm placement within the abdominal aorta, real-time blood pressure trace was assessed using NOTOCORD-hem software (Instem, Staffordshire, UK). As the aorta is the major arterial vessel emerging from the heart, it provides accurate information about HR and blood pressure. Nonabsorbable sutures were used to attach the transmitter unit to the inner musculature of the abdominal wall. Finally, the animal is sewed up with absorbable sutures, provided pain relieving drugs (carprofen 5mg/kg and buprenorphine 0.03mg/kg), and placed in their homecage for recovery.

Suppl. Figure Legends

Suppl.Fig.1. No average behavioral differences between drinkers and non-drinkers, in **(A)** food intake ($Fs<1.56$, $ps>0.2$), **(B)** latency to approach food (log, $Fs<2.3$, $ps>0.13$), **(C)** number

of approaches (Fs<2.35, ps>0.13), **(D)** time in center (log, Fs<1.99, ps>0.16), or **(E)** latency to grab food (log, Fs<0.9, ps>0.3).

Suppl.Fig.2. No average HR/HRV differences between drinkers and non-drinkers for nearly all measures. (A-C) HR **(A)** at baseline (sex: $F_{(1,46)}$=16.17, p=0.0002, other Fs<0.9, ps>0.3), **(B)** during NSF (sex: $F_{(1,46)}$=10.96, p=0.0018, other Fs<1.1, ps>0.3), and **(C)** % change from basal to NSF (log, sex: $F_{(1,46)}$=6.399, p=0.0149, other Fs<0.3, ps>0.6). **(E-G)** SDNN **(E)** at baseline (log, sex: $F_{(1,46)}$=22.08, p<0.0001, other Fs<0.7, ps>0.4), **(F)** during NSF (log, Fs<1.5, ps>0.2) , and **(G)** % change from basal to NSF (log, sex: $F_{(1,46)}$=10.90, p=0.0019, other Fs<0.7, ps>0.4). **(H-J)** rMSSD **(H)** at baseline (log, sex: $F_{(1,46)}$=3.645, p=0.0624, other Fs<0.2, ps>0.7), **(I)** during NSF (log, Fs<1.5, ps>0.2), and **(J)** % change from basal to NSF (log, Fs<2.7, ps>0.11). **(K-M)** SDNN/rMSSD **(K)** at baseline (log, sex: $F_{(1,46)}$=27.12, p<0.0001, other Fs<1.97, ps>0.18), **(L)** during NSF (log, sex: $F_{(1,46)}$=4.246, p=0.0450, other Fs<0.8, ps>0.4), and **(M)** % change from basal to NSF (log, sex: $F_{(1,46)}$=13.51, p=0.0006; interaction: $F_{(1,46)}$=5.289, p=0.0260; drinker-vs-naïve: $F_{(1,46)}$=0.885, p=0.3518). Thus, there was an effect of drinking condition for percent change in SDNN/rMSDD, although with multiple corrections, this would not be considered significant.

We note that, when comparing drinkers and controls in humans, some studies find no HR differences (6,7) or higher HR in drinkers (8-11), and many observe lower basal HFHRV with AUD (7,8,12-14), although with some considerations. One study (10) found no AUD vs control differences in rMSSD, HFHRV, or LFHRV, but did observe greater average entropy with AUD (a non-linear HRV measure). In addition, moderate to heavy drinkers (non-AUD) can have higher HFHRV (15), and resting HRV is greater in people drinking lower levels of alcohol (1–2 drinks/day), but reduced in people consuming more than that (13). One possibility is that some human studies may reflect more advanced AUD stages. For example, Hwang and colleagues (10) noted no HRV changes with alcohol cues, while another study (16) found rMSSD increases to alcohol cues associated with more alcohol problems. However, Hwang et al. (10) noted that AUDIT scores in their study were ~19, but 10-12 in (16). Thus, HRV changes may vary with the level of drinking problems, and our rats would not reflect the highest-level problem drinkers.

Suppl.Fig.3. Raw data and scatter plots for basal and NSF HR/HRV measures.

Also, we ran two-way ANOVAs on the basal-vs-NSF HRV measures, even though some groups for each measure were not normal, to compare basal versus NSF measures (within-subject), and across females and males. Results for SDNN and SDNN/rMSSD were similar to log-normalized data (**Fig.2**). For SDNN, there was a significant effect of sex ($F_{(1,48)}$=22.19, p<0.0001), basal versus NSF ($F_{(1,48)}$=127.1, p<0.0001), and interaction ($F_{(1,48)}$=5.961, p=0.0184). Thus, female basal SDNN was lower than males, and males had a greater drop in SDNN than females. However, there were no significant changes for rMSSD (sex: $F_{(1,48)}$=0.984, p=0.3262; basal-NSF: $F_{(1,48)}$=3.659, p=0.0618; interaction: $F_{(1,48)}$=1.621, p=0.2091). Even so, SDNN/rMSSD showed a significant effect of sex ($F_{(1,48)}$=20.91, p<0.0001), basal versus NSF ($F_{(1,48)}$=103.3, p<0.0001), and interaction ($F_{(1,48)}$=17.56, p=0.0001).

Suppl.Fig.4. First approach latency. With significant effects for both rMSSD and SDNN, there was no association between latency to first approach and SDDN/rMSSD, **(A)** at baseline (females: $F_{(1,20)}$=0.123, R^2=0.006, p=0.7294; males; $F_{(1,25)}$=0.008, R^2=0.000, p=0.9309), or **(B)**

during NSF (females: $F_{(1,20)}$=0.267, R^2=0.013, p=0.6110; males: $F_{(1,25)}$=2.651, R^2=0.096, p=0.1160).

Suppl.Fig.5. Examining whether higher HR was associated with reduced HRV, a mathematical relationship which could impact HRV interpretations. **(A-C)** For baseline HR measures, female HR (and trends in males) was associated with **(A)** lower SDNN (female $F_{(1,21)}$=37.11, R^2=0.639, p<0.0001; male $F_{(1,25)}$=3.514, R^2=0.123, p=0.0726), **(B)** lower rMSSD (female $F_{(1,21)}$=27.49, R^2=0.567, p<0.0001; male $F_{(1,25)}$=4.218, R^2=0.144, p=0.0506), and **(C)** lower SDNN/rMSSD ratio (female $F_{(1,21)}$=4.632, R^2=0.181, p=0.0432; male $F_{(1,25)}$=1.227, R^2=0.047, p=0.2785). **(D-F)** For NSF HR measures, both sexes had significantly lower HRV with higher HR, including for **(D)** SDNN (female $F_{(1,21)}$=8.368, R^2=0.285, p=0.0087; male $F_{(1,25)}$=21.96, R^2=0.468, p<0.0001) and **(E)** rMSSD (female $F_{(1,21)}$=12.55, R^2=0.374, p=0.0019; male $F_{(1,25)}$=20.88, R^2=0.455, p<0.0001), but not **(F)** SDNN/rMSSD (female $F_{(1,21)}$=2.606, R^2=0.110, p=0.1214; male $F_{(1,25)}$=3.308, R^2=0.117, p=0.0809) which may be due to concurrent decreases in both SDNN and rMSSD. Together, data in **(A-F)** suggest that HRV was lower under conditions with higher HR, females at baseline and NSF, and males during NSF. **(G-I)** Even so, basal HR did not correlate with **(G)** the change in SDNN (NSF minus basal, female $F_{(1,21)}$=0.954, R^2=0.043, p=0.3398; male $F_{(1,25)}$=3.226, R^2=0.114, p=0.0846), or **(H)** change in rMSSD, although a trend in males (female $F_{(1,21)}$=0.035, R^2=0.002, p=0.8539; male $F_{(1,25)}$=3.835, R^2=0.133, p=0.0614), and where **(I)** higher basal HR correlated with smaller change in SDNN/rMSSD in females ($F_{(1,21)}$=4.812, R^2=0.186, p=0.0397) but not males ($F_{(1,25)}$=0.666, R^2=0.026, p=0.4221). Thus, these data support the possibility that higher HR was associated with reduced HRV, which might impact HRV patterns seen with latency to first approach food (**Fig.4**). On the other hand, results in **(G,H)** suggest that there was some dynamic range for HRV measures to change, separate from basal HR. *,**,*** p<0.05, p<0.01, *** p<0.001.

Suppl.Fig.6. HR: number of approaches and time in center. HR did not relate to **(A,B)** number of approaches **(A)** at baseline (males: $F_{(1,25)}$=1.824, R^2=0.068, p=0.1889; females: $F_{(1,19)}$=3.511, R^2=0.156, p=0.0764) or **(B)** during NSF (males: $F_{(1,25)}$=0.159, R^2=0.006, p=0.6939; females: $F_{(1,19)}$=0.270, R^2=0.014, p=0.6092). **(C,D)** HR was also not correlated with time in center **(C)** at baseline (males: $F_{(1,25)}$=0.732, R^2=0.028, p=0.4005; females: $F_{(1,19)}$=0.053, R^2=0.003, p=0.8205) or **(D)** during NSF (males: $F_{(1,25)}$=1.826, R^2=0.068, p=0.1887; females: $F_{(1,19)}$=0.991, R^2=0.050, p=0.3320).

Suppl.Fig.7. Males with a larger change in SDNN/rMSSD from baseline to NSF had more approaches ($F_{(1,25)}$=5.938, R^2=0.192, p=0.0223), which was not observed in females ($F_{(1,19)}$=1.095, R^2=0.055, p=0.3085).

Suppl.Fig.8. Relation between different NSF behaviors. (A) After removing a male outlier (600s latency to approach), there was no relation between food intake and latency to first approach in females ($F_{(1,20)}$=0.596, R^2=0.029, p=0.4491) or males ($F_{(1,25)}$=2.201, R^2=0.081, p=0.1504). **(B)** No relation between food intake and number of approaches in females ($F_{(1,19)}$=0.697, R^2=0.035, p=0.4142) or males ($F_{(1,25)}$=0.714, R^2=0.028, p=0.4063). **(C)** Food intake was significantly and negatively correlated with latency to first grab food in females ($F_{(1,19)}$=21.94, R^2=0.536, p=0.0002) and males ($F_{(1,25)}$=55.64, R^2=0.690, p<0.0001). However, no HR/HRV measure correlated with latency to grab food (not shown). *** p<0.001.

Suppl.Fig.9. rMSSD measures across the session, centered on the time to grab food. See Figure 9 legend for details. No differences across analysis time points for rMSSD (**C,D,** female: Friedman stat=1.444, p=0.4857, male: Friedman stat=0.947, p=0.9474).

Suppl.Fig.10-14. In these figures, we show correlations between different NSF behavioral measures and the log transformation of each HR/HRV measures. Overall, sex and behavior differences were similar to those described in the manuscript using the raw values of the different HR/HRV measures. Statistical testing is described in the figure for each panel.

References

1. Darevsky D, Gill TM, Vitale KR, Hu B, Wegner SA, Hopf FW. Drinking despite adversity: behavioral evidence for a head down and push strategy of conflict-resistant alcohol drinking in rats. *Addict Biol*. (2019) 24:426-37.
2. Darevsky D Hopf FW. Behavioral indicators of succeeding and failing under higher-challenge compulsion-like alcohol drinking in rat. *Behav Brain Res*. (2020) 393:112768.
3. Seif T, Chang SJ, Simms JA, Gibb SL, Dadgar J, Chen BT et al. Cortical activation of accumbens hyperpolarization-active NMDARs mediates aversion-resistant alcohol intake. *Nat Neurosci*. (2013) 16:1094-100.
4. De Oliveira Sergio T, Lei K, Kwok C, Ghotra S, Wegner SA, Walsh M et al. The role of Anterior Insula-brainstem projections and alpha-1 noradrenergic receptors for compulsion-like and alcohol-only drinking. *Neuropsychopharmacology*. (2021) 35:1751-1760.
5. Gobinath AR, Wong S, Chow C, Lieblich SE, Barr AM, Galea LAM. Maternal exercise increases but concurrent maternal fluoxetine prevents the increase in hippocampal neurogenesis of adult offspring. *Psychoneuroendocrinology*. (2018) 91:186-197.
6. Rajan I, Murthy PJ, Ramakrishnan AG, Gangadhar BN, Janakiramaiah N (1998): Heart rate variability as an index of cue reactivity in alcoholics. *Biol Psychiatry* 43: 544-6.
7. Romero-Martinez A, Vitoria-Estruch S Moya-Albiol L (2019): Emotional and autonomic dysregulation in abstinent alcoholic men: An idiosyncratic profile? *Alcohol* 77: 155-162.
8. Ingjaldsson JT, Laberg JC Thayer JF (2003): Reduced heart rate variability in chronic alcohol abuse: relationship with negative mood, chronic thought suppression, and compulsive drinking. *Biol Psychiatry* 54: 1427-36.
9. Seo D, Lacadie CM, Tuit K, Hong KI, Constable RT, Sinha R (2013): Disrupted ventromedial prefrontal function, alcohol craving, and subsequent relapse risk. *JAMA Psychiatry* 70: 727-39.
10. Hwang S, Martins JS, Douglas RJ, Choi JJ, Sinha R, Seo D (2022): Irregular Autonomic Modulation Predicts Risky Drinking and Altered Ventromedial Prefrontal Cortex Response to Stress in Alcohol Use Disorder. *Alcohol Alcohol* 57: 437-444.
11. Sinha R, Fox HC, Hong KA, Bergquist K, Bhagwagar Z, Siedlarz KM (2009): Enhanced negative emotion and alcohol craving, and altered physiological responses following stress and cue exposure in alcohol dependent individuals. *Neuropsychopharmacology* 34: 1198-208.
12. Quintana DS, Guastella AJ, McGregor IS, Hickie IB, Kemp AH (2013): Heart rate variability predicts alcohol craving in alcohol dependent outpatients: further evidence for

HRV as a psychophysiological marker of self-regulation. *Drug Alcohol Depend* 132: 395-8.
13. Karpyak VM, Romanowicz M, Schmidt JE, Lewis KA, Bostwick JM (2014): Characteristics of heart rate variability in alcohol-dependent subjects and nondependent chronic alcohol users. *Alcohol Clin Exp Res* 38: 9-26.
14. Cheng YC, Huang YC Huang WL (2019): Heart rate variability as a potential biomarker for alcohol use disorders: A systematic review and meta-analysis. *Drug Alcohol Depend* 204: 107502.
15. Mayhugh RE, Laurienti PJ, Fanning J, Gauvin L, Heilman KJ, Porges SW *et al.* (2018): Cardiac vagal dysfunction moderates patterns of craving across the day in moderate to heavy consumers of alcohol. *PLoS One* 13: e0200424.
16. Wang W, Zhornitsky S, Le TM, Zhang S, Li CR (2020): Heart Rate Variability, Cue-Evoked Ventromedial Prefrontal Cortical Response, and Problem Alcohol Use in Adult Drinkers. *Biol Psychiatry Cogn Neurosci Neuroimaging* 5: 619-628.

Frasier et al. Fig.S1

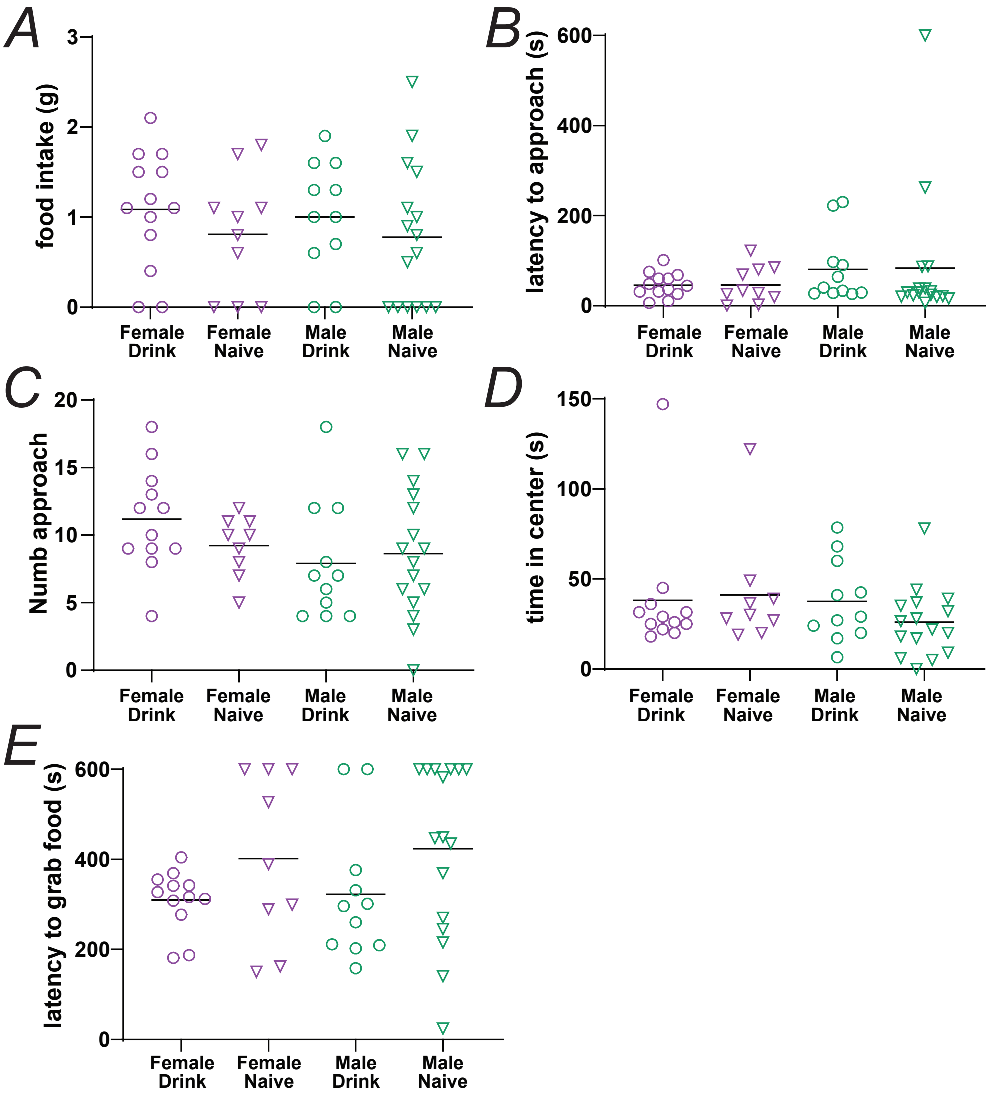

Frasier et al. Fig.S2

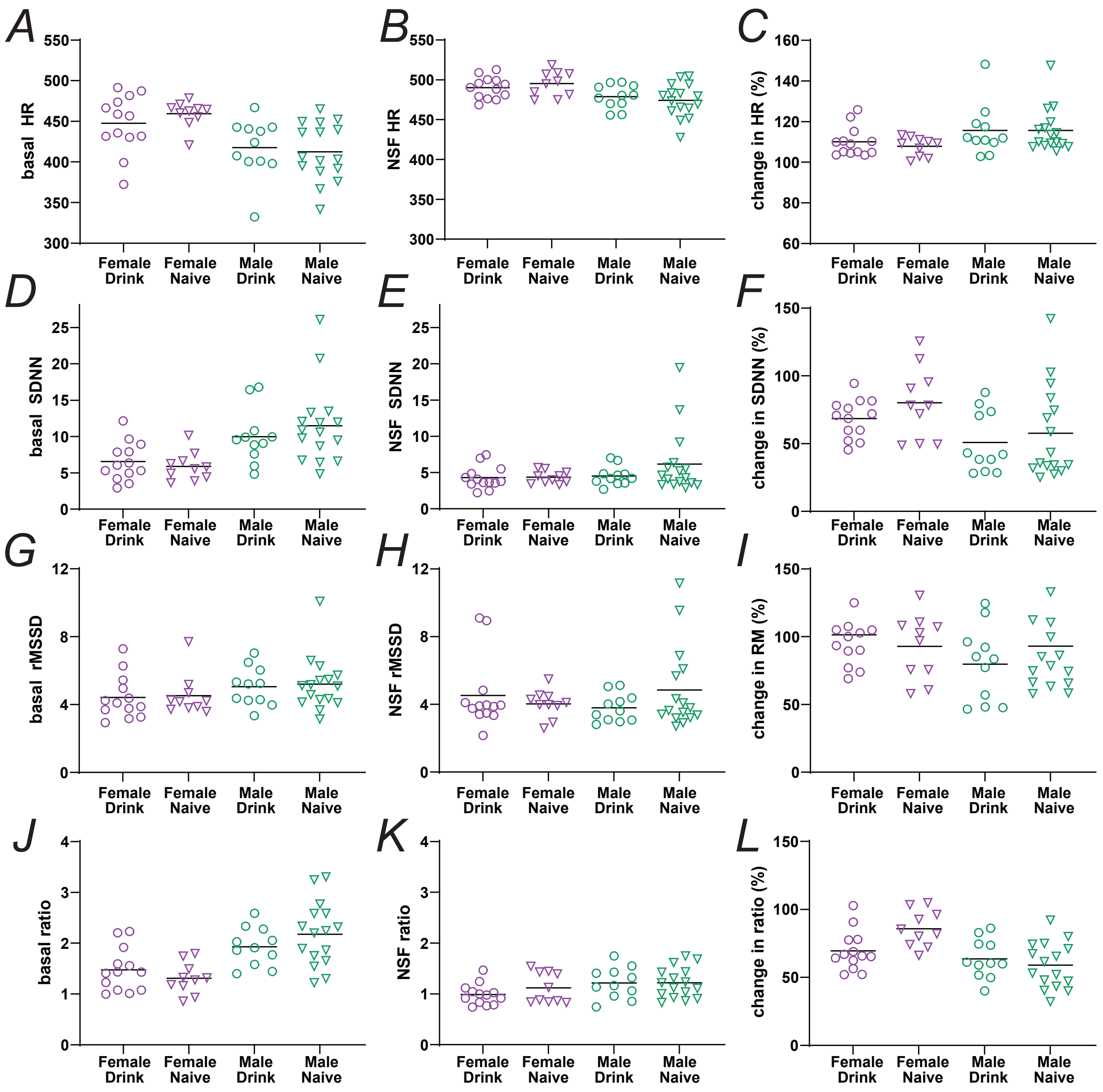

Frasier et al. Fig.S3

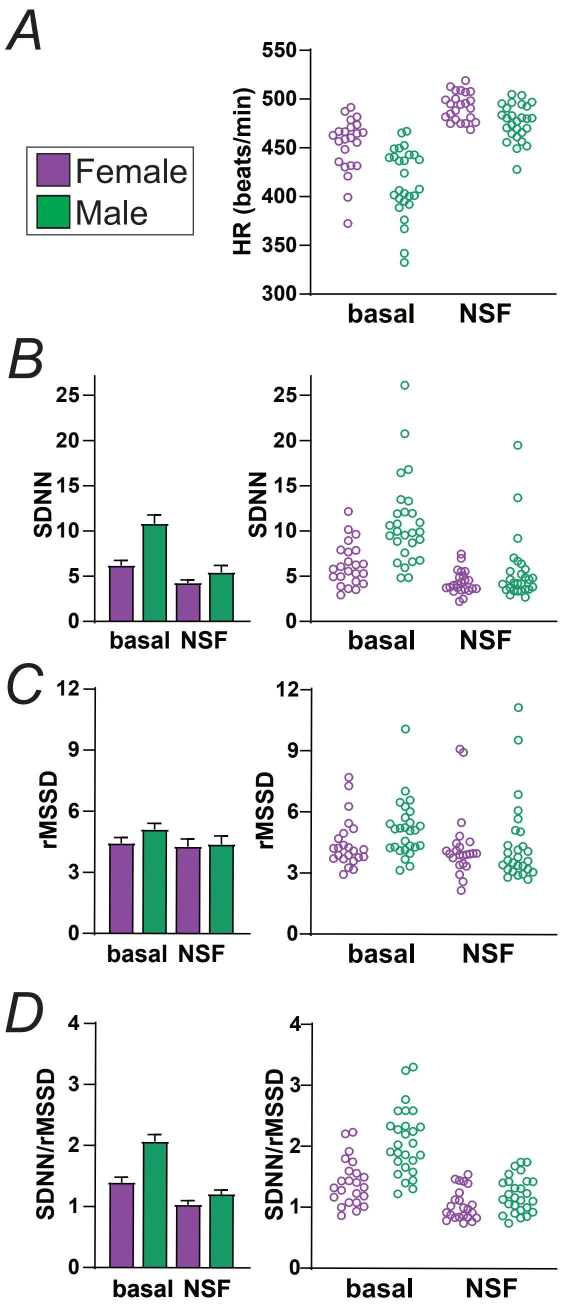

Frasier et al., Fig.S4

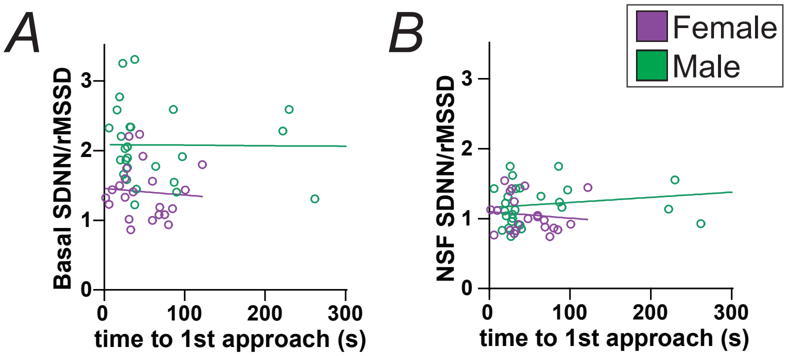

Frasier et al., Fig.S5

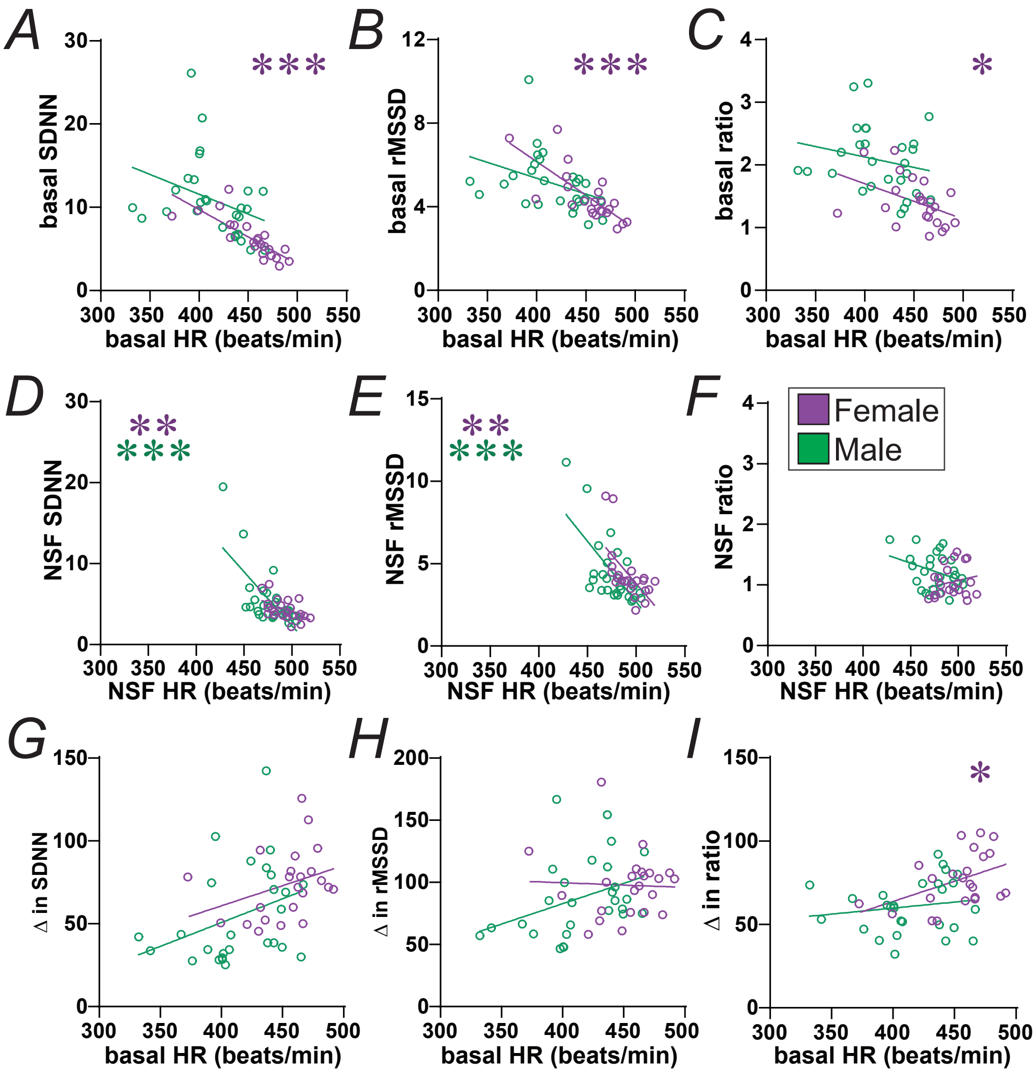

Frasier et al., Fig.S6

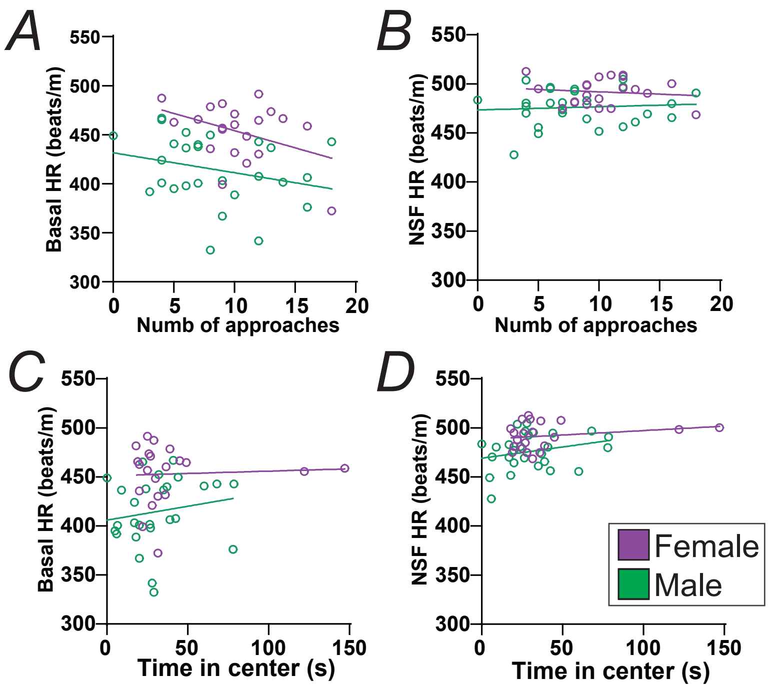

Frasier et al., Fig.S7

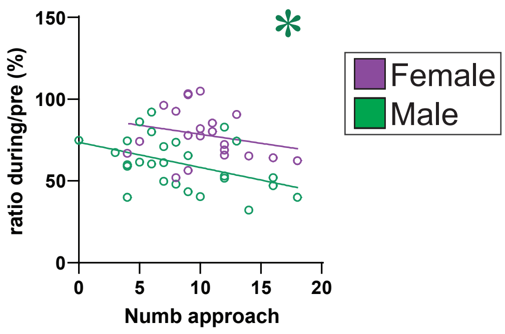

Frasier et al. Fig.S8

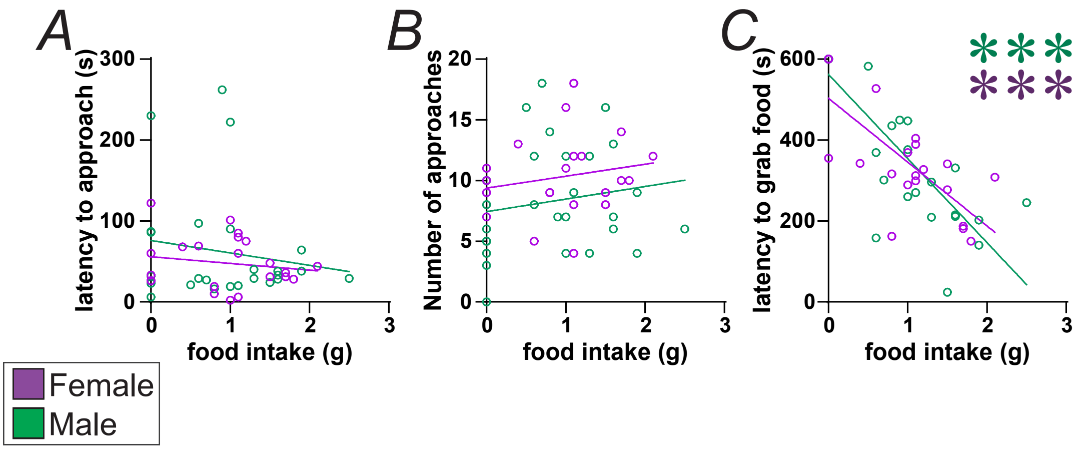

Frasier et al. Fig.S9

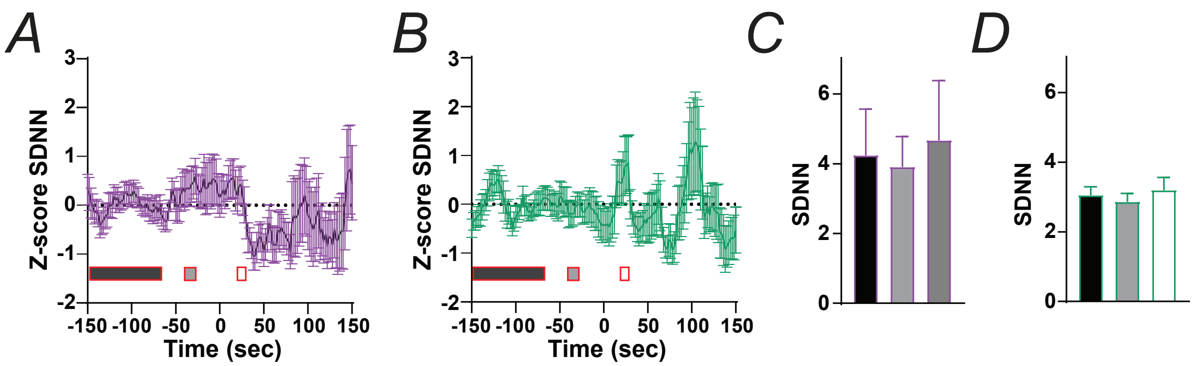

Frasier et al., Fig.S10

Log HR/HRV data for Food Intake

female F=4.425, p=0.0476; male F=1.412, p=0.2460; p=0.0270 diff in slopes

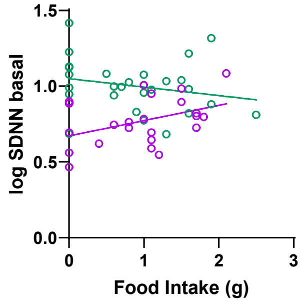

female p=0.9150; male p=0.5367

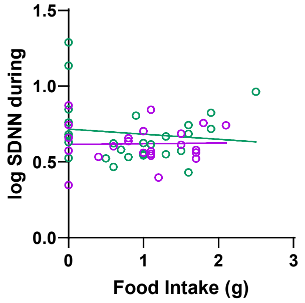

female F=6.136, p=0.0218; male F=0.305, p=0.5854; p=0.0734 diff in slopes

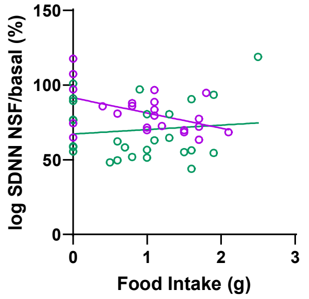

female p=0.2665; male p=0.8215

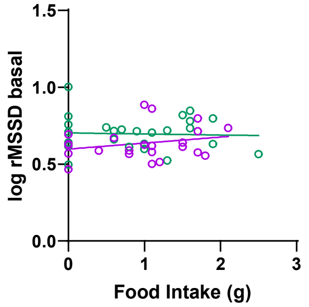

female p=0.9015; male p=0.9687

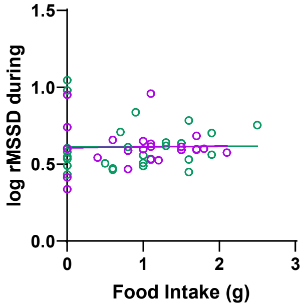

female p=0.5637; male p=0.5345

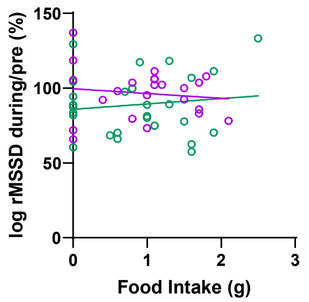

female p=0.0880; male p=0.1197

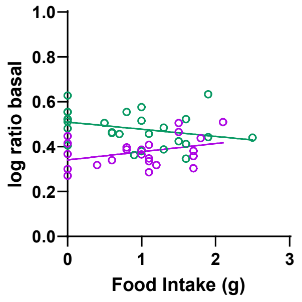

female p=0.9880; male p=0.2139

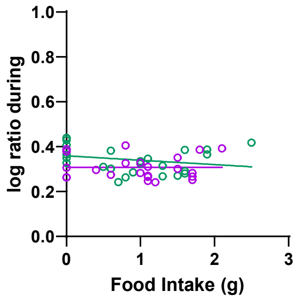

female F=5.977, p=0.0234; male F=0.099, p=0.7560, p=0.0590 diff in slopes

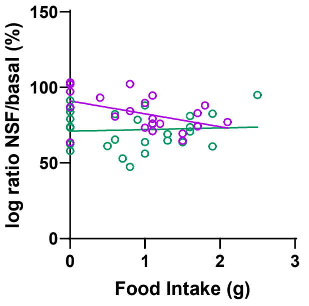

female p=0.2948; male p=0.6665

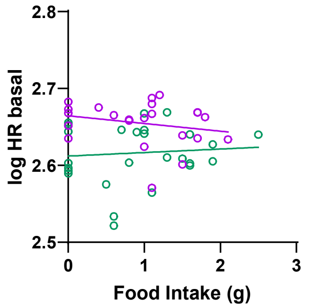

female p=0.4689; male p=0.4693

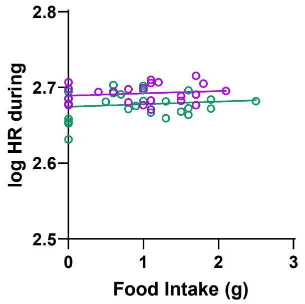

female F=6.136, p=0.0218; male F=0.305, p=0.5854, p=0.0734 diff in slopes, p=0.0181 diff in intercepts

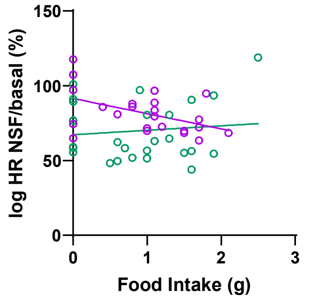

Frasier et al., Fig.S11

Log HR/HRV data for Time to First Approach

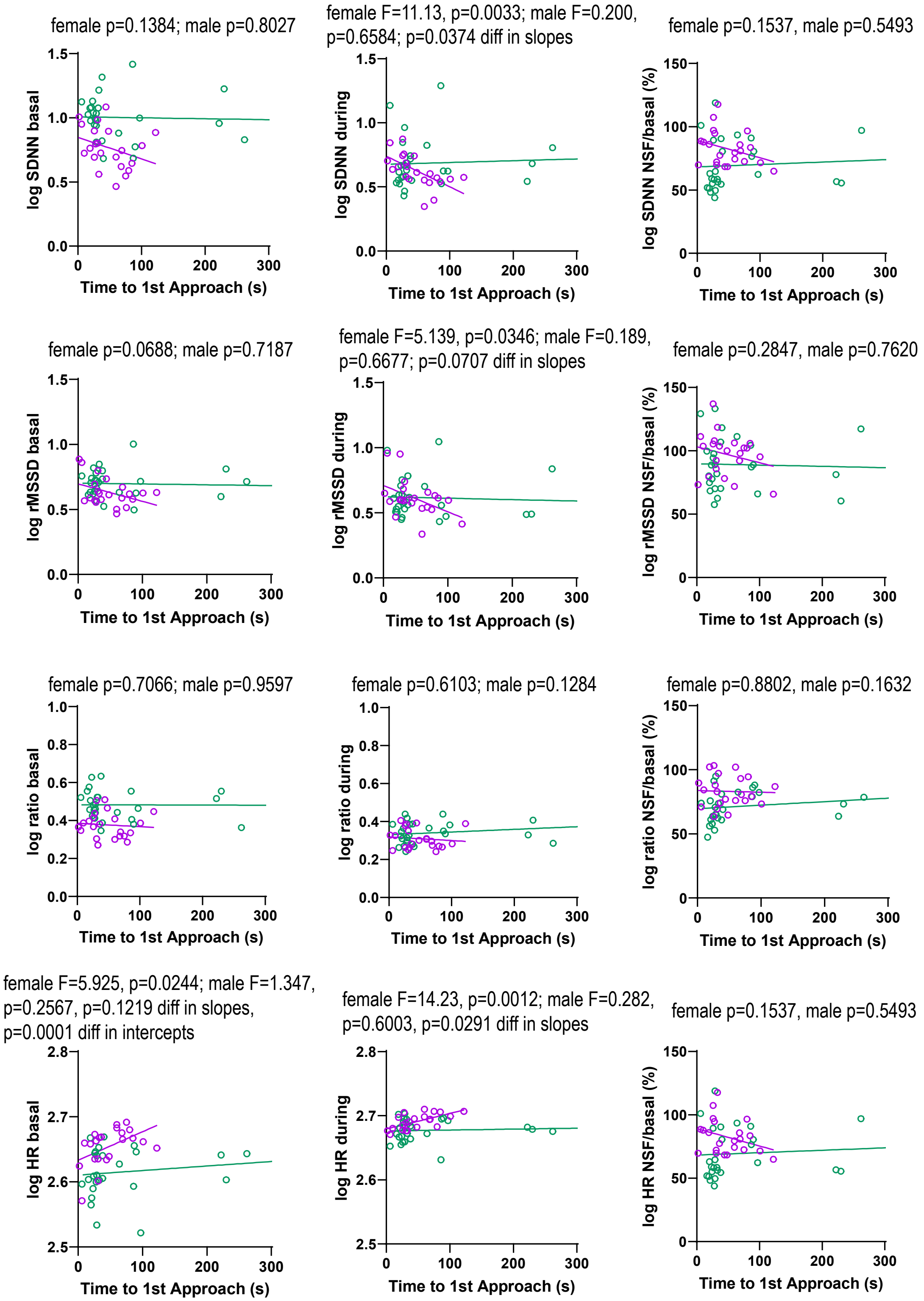

Frasier et al., Fig.S12

Log HR/HRV data for Number of Approaches

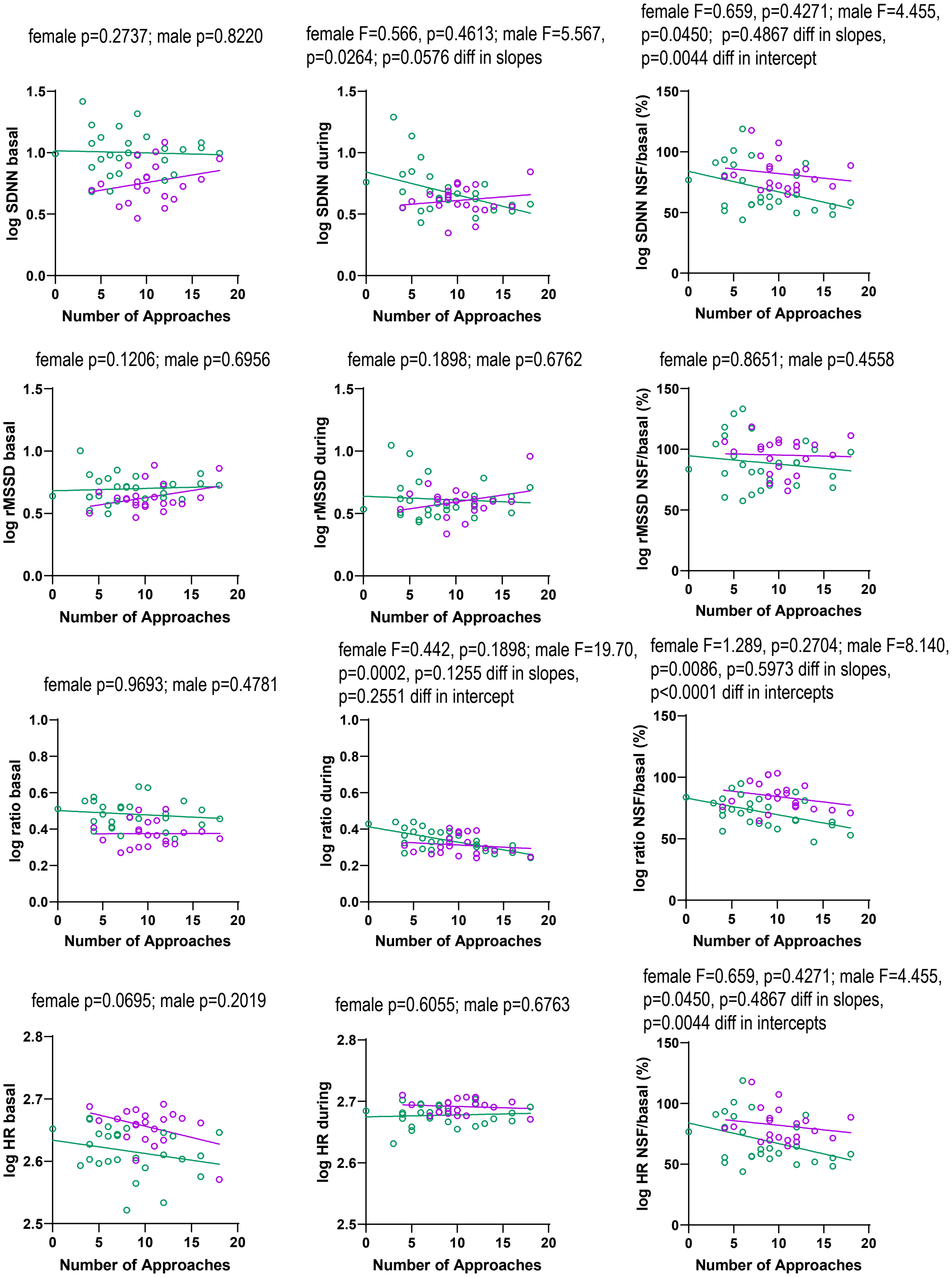

Frasier et al., Fig.S13

Log HR/HRV data for Time in Center

female p=0.8650; male p=0.0983

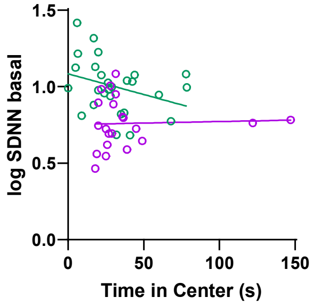

female F=0.000, p=0.9871; male F=5.186, p=0.0316; p=0.0402 diff in slopes

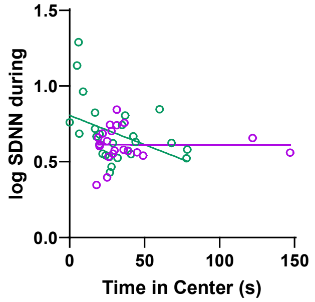

female p=0.6995, male p=0.3301

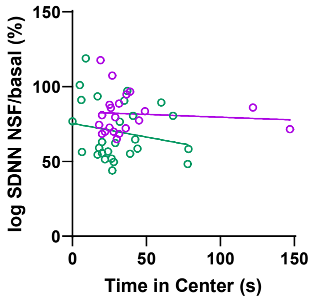

female p=0.8890; male p=0.4538

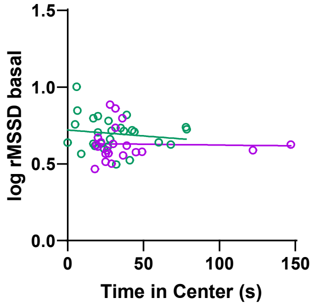

female p=0.6428; male p=0.4120

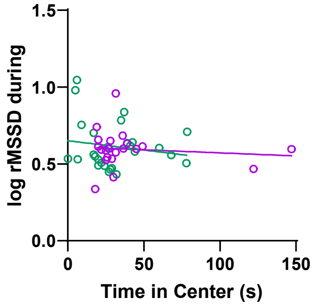

female p=0.6084, male p=0.6929

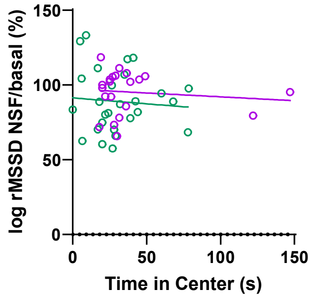

female p=0.7807; male p=0.0636

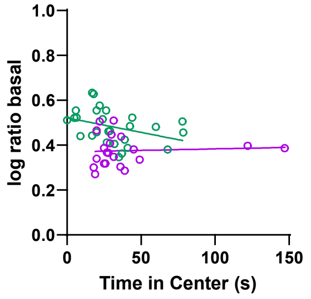

female F=0.343, p=0.5653; male F=9.215, p=0.0055, p=0.0090 diff in slopes

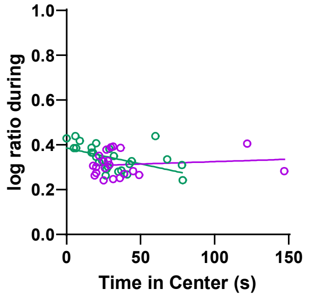

female p=0.9200, male p=0.3837

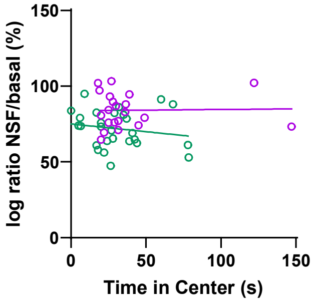

female p=0.7925; male p=0.4225

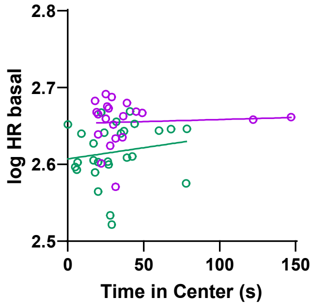

female p=0.3275; male p=0.1842

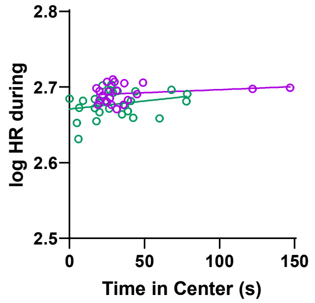

female p=0.6995, male p=0.3301

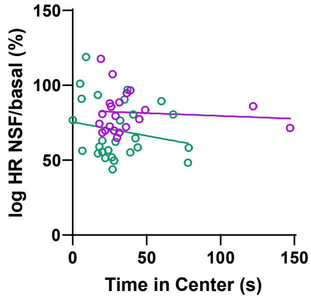

Supplement: Supplementary file 1 [file Data_Sheet_1.pdf]
